# The Influence of Climate, Atmospheric Pollution, and Natural Disasters on Cardiovascular Diseases and Diabetes Mellitus in Drylands: A Scoping Review

**DOI:** 10.3389/phrs.2024.1607300

**Published:** 2024-08-08

**Authors:** Rafaella Pessoa Moreira, Clara Beatriz Costa da Silva, Tainara Chagas de Sousa, Flávia Lavinnya Betsaida Félix Leitão, Huana Carolina Cândido Morais, Andressa Suelly Saturtino de Oliveira, Gonzalo Duarte-Clíments, María Begoña Sánchez Gómez, Tahissa Frota Cavalcante, Alexandre Cunha Costa

**Affiliations:** ^1^ Institute of Health Sciences, University of International Integration of Afro-Brazilian Lusophony, Redenção, Brazil; ^2^ School of Nursing, University of La Laguna, San Cristóbal de La Laguna, Spain; ^3^ School of Nursing, Valencian International University, Castelló de la Plana, Spain; ^4^ Department of Nursing, UCAM Catholic University of Murcia, Guadalupe, Spain; ^5^ Institute of Engineering and Sustainable Development, University of International Integration of Afro-Brazilian Lusophony, Redenção, Brazil

**Keywords:** climate change, atmospheric pollution, environmental resilience, cardiovascular diseases, diabetes mellitus

## Abstract

**Objectives:**

In the face of escalating global aridification, this study examines the complex relationship between climate variability, air pollution, natural disasters, and the prevalence of cardiovascular disease (CVD) and diabetes mellitus (DM) in arid regions.

**Methods:**

The study conducted a scoping review of multiple databases using JBI guidelines and included 74 studies.

**Results:**

The results show that acute myocardial infarction (n = 20) and stroke (n = 13) are the primary CVDs affected by these factors, particularly affecting older adults (n = 34) and persons with hypertension (n = 3). Elevated air temperature and heat waves emerge as critical risk factors for CVD, exacerbating various cardiovascular mechanisms. Atmospheric pollutants and natural disasters increase this risk. Indirect effects of disasters amplify risk factors such as socioeconomic vulnerability (n = 4), inadequate medical care (n = 3), stress (n = 3), and poor diet (n = 2), increasing CVD and DM risk.

**Conclusion:**

The study underscores the need for nations to adhere to the Paris Agreement, advocating for reduced air pollutants, resilient environments, and collaborative, multidisciplinary research to develop targeted health interventions to mitigate the adverse effects of climate, pollution, and natural disasters.

## Introduction

Climate change is characterized by long-term increase in temperature, changes in the geographical distribution of precipitation, and an increase in the magnitude and frequency of extreme weather events such as floods, droughts, and heatwaves [1, 2]. These changes in weather patterns can influence people’s health and lead to the development of Climate-Sensitive Diseases (CSD) [3, 4]. Climate change represents one of the most significant threats to human wellbeing [5], making the global population more vulnerable to CSDs such as cardiovascular diseases (CVD) and diabetes mellitus (DM) [6–9].

In the face of climate change, climate projections indicate an increase of drylands worldwide over the coming decades [10–12]. Drylands (arid, semi-arid, and dry sub-humid lands) are characterized by natural water scarcity, where the annual ratio between precipitation and evapotranspiration, or aridity index, is greater than 0.05 and less than 0.65. Drylands cover 41% of the land surface, produce 44% of the crops, and contain over 2 billion people and half of the world’s livestock [13].

In dry regions, extreme weather events such as droughts [14], dust or sandstorms [15], heatwaves [16], and/or fires are frequent [12, 17, 18]. These weather events can occur in combination [19]. Additionally, other natural disasters such as earthquakes and hurricanes may also be recurrent and affected by climate change [20]. Populations in drylands, constantly affected by natural disasters, are more vulnerable to subsistence crises [21], poverty, economic and political marginalization [22], and health problems [23].

Recent studies highlight the effects of climate on cardiovascular diseases [24] and diabetes mellitus [25], where extreme temperatures, changes in relative humidity, and wind intensity were determining factors. Furthermore, the role of global climate phenomena such as El Niño in the development and/or exacerbation of CVD is emphasized [24–26].

Air pollution, combined with climatic effects, poses an additional risk factor for mortality from cardiovascular diseases and influences the increase in hospitalizations due to diabetes mellitus [27, 28]. Furthermore, the contribution of increased air pollution to the retention of greenhouse gases, responsible for warming the Earth’s surface and raising temperatures globally [1], highlights its role in the long-term effects of climate change on CVD.

Thus, in addition to established risk factors for CVD and DM, known as non-modifiable (ethnicity, age, family history) and modifiable (sedentary lifestyle, poor diet, alcoholism, smoking, dyslipidemia, obesity) [29, 30], it is necessary to map the risk factors for CVD and DM related to climate variability, air pollution, and natural disasters. In this context, it is also relevant to identify populations living in dry regions that are most vulnerable to developing cardiovascular diseases and diabetes mellitus.

The general objective of this scoping review was to map the influence of climate variability, air pollution, and natural disasters on cardiovascular diseases and diabetes mellitus in drylands, aiming to identify the main cardiovascular diseases, the most vulnerable populations, the socio-environmental factors related to physiological mechanisms for the development and/or exacerbation of these diseases, and the main risk factors intensified by natural disasters.

It is expected that the results of this research will be useful for the development of public health protocols that can guide the implementation of effective multidisciplinary interventions for the prevention of CVD and DM and their complications resulting from climate variability, air pollution, and natural disasters in dry regions, especially in populations identified as most vulnerable in a context of climate change.

## Methods

A scoping review was conducted following the Joanna Briggs Institute (JBI) approach for scoping reviews, the PRISMA Extension for Scoping Reviews (PRISMA-ScR) guidelines, and the JBI Evidence Synthesis Manual. The protocol was registered in the Open Science Framework (OSF). Registration DOI.

### Review Question

The Population, Concept, and Context (PCC) framework was adopted to formulate the research questions:

#### Population

Individuals with cardiovascular diseases: Group of heart and blood vessel diseases, including: coronary artery disease, acute myocardial infarction; cerebrovascular disease: peripheral artery disease; rheumatic heart disease; congenital heart disease; deep vein thrombosis and pulmonary embolism [31]; diabetes: Chronic metabolic disease characterized by high blood glucose levels, leading over time to serious damage to the heart, blood vessels, eyes, kidneys, and nerves [31].

#### Concept

Climate variability: Variations in climate compared to its average, resulting in changes in air pressure, temperature, precipitation, humidity, and other climate variables [32]. The climate variables included in this study are: air temperature, precipitation, air humidity, wind speed, and phenomena such as El Niño; Air pollution: Refers to contamination of the indoor or outdoor environment resulting in the modification of the atmosphere’s natural characteristics. This contamination can occur due to chemical, physical, or biological agents [20]; Natural disasters: Severe weather conditions, including floods, tornadoes, hurricanes, wildfires, droughts, earthquakes, or a combination of these extreme events. They represent a major threat to infrastructure, health, property, and human security [33].

#### Context

Drylands characterized by water scarcity (a ratio between average annual precipitation and potential evapotranspiration, also known as aridity index, greater than 0.05 and less than 0.65). They can be classified as arid, semi-arid, and dry sub-humid lands [34].

Based on the aforementioned strategy, the following research question was formulated: 1) What is the influence of climate variability, air pollution, and natural disasters on cardiovascular diseases and diabetes mellitus in drylands? In this context, the following auxiliary questions were identified: 1) What are the most prevalent cardiovascular diseases and which populations are most vulnerable? 2) What are the effects of exposure to climate variability, air pollution, and natural disasters on hospitalizations and deaths from cardiovascular diseases? 3) What are the socio-environmental factors related to physiological mechanisms for the development and/or exacerbation of cardiovascular diseases? 4) What are the main risk factors for cardiovascular diseases and diabetes mellitus intensified by natural disasters?

### Eligibility Criteria

Inclusion: Studies that addressed the research questions, formulated based on the PCC framework, were included. Multinational studies that included any dry climate region were also included in this review.

Exclusion: Descriptive studies without identification of influencing factors, duplicate studies (only one of the studies was considered for analysis), and studies not available in full text.

### Study Designs

We included studies using various designs: experimental and quasi-experimental studies (randomized and non-randomized controlled trials), before-after and interrupted time series studies, and analytical observational studies (prospective and retrospective cohort studies, case-control studies, and cross-sectional analytical studies), as well as descriptive observational studies including case series and reports of individual cases, qualitative studies, and systematic reviews. No language or time restrictions were applied.

### Search Strategy

The search strategies were developed by the research team in collaboration with a librarian, utilizing the PCC components. Search terms were identified from controlled vocabularies such as Health Sciences Descriptors (DeCS), Medical Subject Headings (MeSH), and Embase Subject Headings (Emtree).

The search involved three steps. Firstly, a preliminary search in PubMed, Cochrane Library, OSF, and JBI Synthesis identified keywords and terms for the search strategy. Secondly, this strategy was applied to all chosen databases. A third search checked reference lists for additional sources. The detailed search strategies are in Supplementary Appendix Table S1. Searches were conducted on 04 September 2023, across various databases and gray literature sources, including Agris, Virtual Health Library, Academic Search Premier, CINAHL, GreenFILE, SocINDEX, CAB Direct, Cochrane Library, Embase, Engineering Village, Epistemonikos, PubMed Central, PubMed, Scielo, Scopus, and Web of Science.

### Descriptors

Cardiovascular diseases; Cardiopathies; Diabetes Mellitus; Stroke; Coronary Disease; Coronary Artery Disease; Droughts; Dryland; Arid Season; Arid Zone; Semi-Arid Zone; “atmosphere pollution”; Natural Disasters.

### Study Selection

After completing the bibliographic search, all results obtained from the databases were grouped and then exported to Rayyan CRI from Qatar, specifically for blinded document selection. Duplicates were removed, and titles and abstracts were independently assessed by two pairs of blinded reviewers based on predefined inclusion criteria. Potentially relevant sources were retrieved in full, and citation details were imported into the JBI System for unified management, assessment, and information review. The full text of selected citations was evaluated according to the inclusion criteria by two pairs of independent reviewers. Reasons for excluding full-text articles were recorded and reported. Any discrepancies among reviewers at each stage of the selection process were resolved through discussion or involvement of a third reviewer—the responsible researcher. Screening was only completed when the agreement proportion among reviewers reached or exceeded 75% [35].

### Methodological Quality Assessment

As this study is a scoping review aiming to map available evidence, no bias risk assessment or quality assessment of included studies was conducted. However, we emphasize that the study remains consistent with the methodology proposed for scoping reviews [35].

### Graphs, Data Extraction, and Synthesis of Results

Data extraction included the following elements: database, title, year, country, authorship, objective, study design, cardiovascular diseases/diabetes mellitus, risk factors, gender, age, and climatic variable. Results were mapped, organized into tables/diagrams/figures, and subjected to thematic analysis and narrative synthesis based on full-text article reading. Labels reflecting content were created for passages that addressed research questions, and similar passages were subsequently grouped [36], resulting in the construction of three thematic categories: climate variability, air pollution, and natural disasters. Figures were created to present thematic categories and associated risk factors influencing the development of CVD or exacerbation of DM, aiming to synthesize the findings of this review. Tables are provided in Supplementary Appendix S1.

### Role of Funding Source

The funding sources had no involvement in study design; in the collection, analysis, and interpretation of data; in the writing of the report; and in the decision to submit the paper for publication.

## Results

**FIGURE 1:**
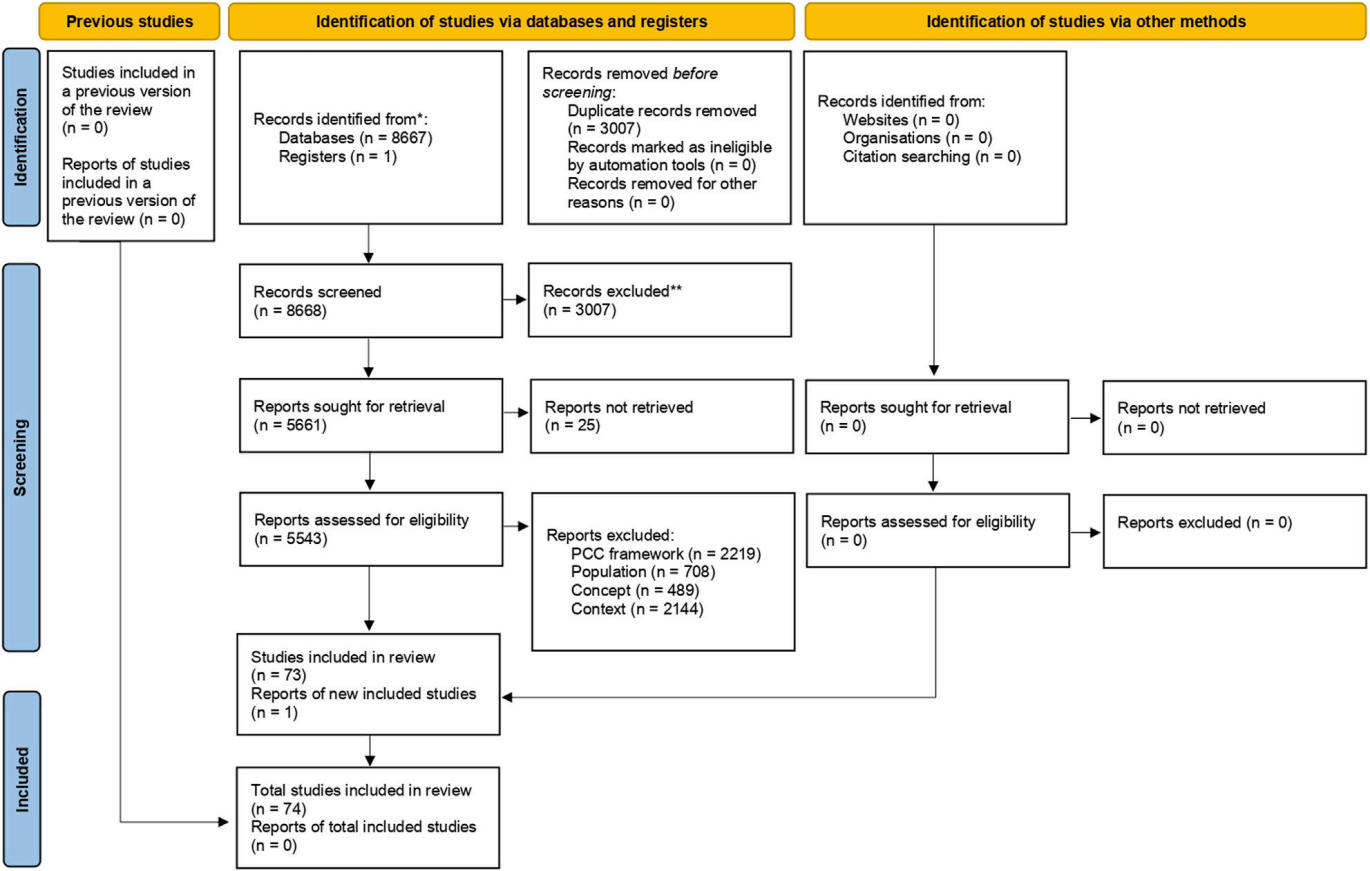
Flowchart of study selection based on the Preferred Reporting Items for Systematic Reviews and Meta-Analyses Extension for Scoping Reviews (2023, Brazil).

The search process is summarized in a PRISMA flow diagram in Figure 1. Initially, a total of 5,660 studies were identified. Following title and abstract screening, 2,180 studies were selected for further consideration. Subsequently, after full-text screening, 228 studies remained. After a second full-text screening, 74 studies were ultimately selected for data extraction as they met the inclusion criteria. A synthesis of the selected studies can be found in Supplementary Appendix S2.

**FIGURE 2:**
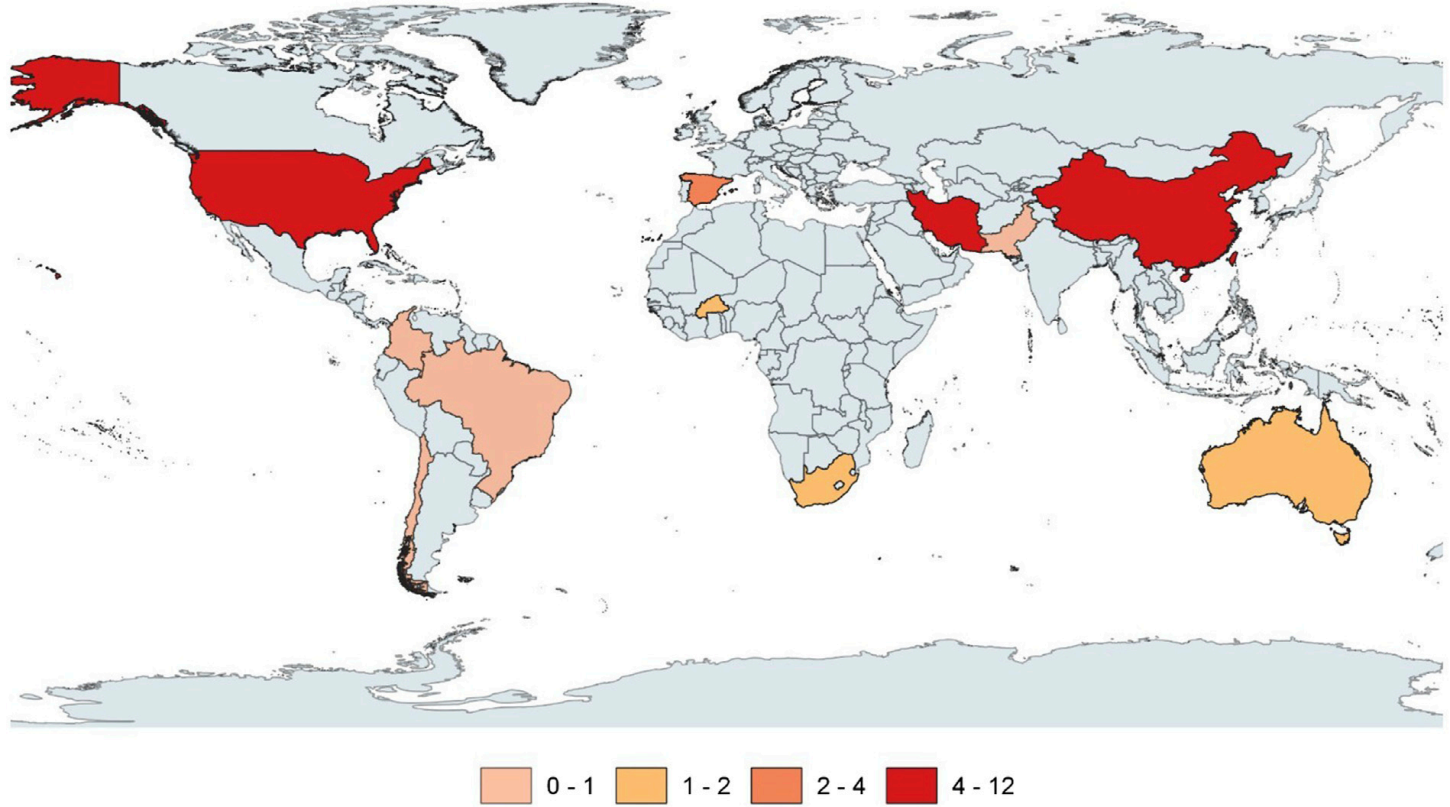
Geographical distribution of publications by country (2023, Brazil). **(A)** Thematic categories. **(B)** Climate variability. **(C)** Atmospheric pollution. **(D)** Natural disasters.

The articles identified in the review are notably recent, with 43 having been published within the last 5 years (2018–2023). The studies were conducted in 12 countries, predominantly high-income (n = 17) and upper-middle-income (n = 16) countries. Only 2 studies were conducted in low-income countries. The income levels of countries were based on the World Bank classification (World Bank Group, 2024). The four countries with the highest number of studies were the United States (n = 12), Iran (n = 12), and China (n = 11). The geographical distribution of the number of publications for each country is depicted in Figure 2 and detailed in Supplementary Appendix Table S2.

A variety of study designs were identified, including time series (n = 14), retrospective (n = 7), systematic reviews (n = 7), narrative reviews (n = 6), ecological (n = 5), literature reviews (n = 4), clinical studies (n = 3), cross-sectional (n = 2), longitudinal (n = 2), cohort (n = 1), cross-sectional case-control (n = 1), regression analysis (n = 1), and report (n = 1). Some studies were categorized by researchers as quantitative (n = 19) and qualitative (n = 4) without specification of study design (Supplementary Appendix Table S3). The studies were retrieved from databases and portals including PubMed/Medline (n = 28), Scopus (n = 24), EBSCO (n = 17), Scielo (n = 2), BVS (n = 1), Embase (n = 1), and Institute of Electric and Electronic Engineers (n = 1) (Supplementary Appendix Table S4; Supplementary Appendix Figure S1).

The cardiovascular diseases identified included acute myocardial infarction (AMI) (n = 20), stroke (n = 13), systemic arterial hypertension (SAH) (n = 8), and heart failure (HF) (n = 3). In addition, diabetes mellitus was identified (n = 12) (Supplementary Appendix Table S5). The most vulnerable populations for CVD and diabetes mellitus in the dry regions were the older adults (n = 34), women (n = 9), and children aged 0–5 years (n = 5). Among the most affected were older adults aged 65–94 years with arterial hypertension (n = 3) (Supplementary Appendix Tables S6, S7; Supplementary Appendix Figure S2).

**FIGURE 3:**
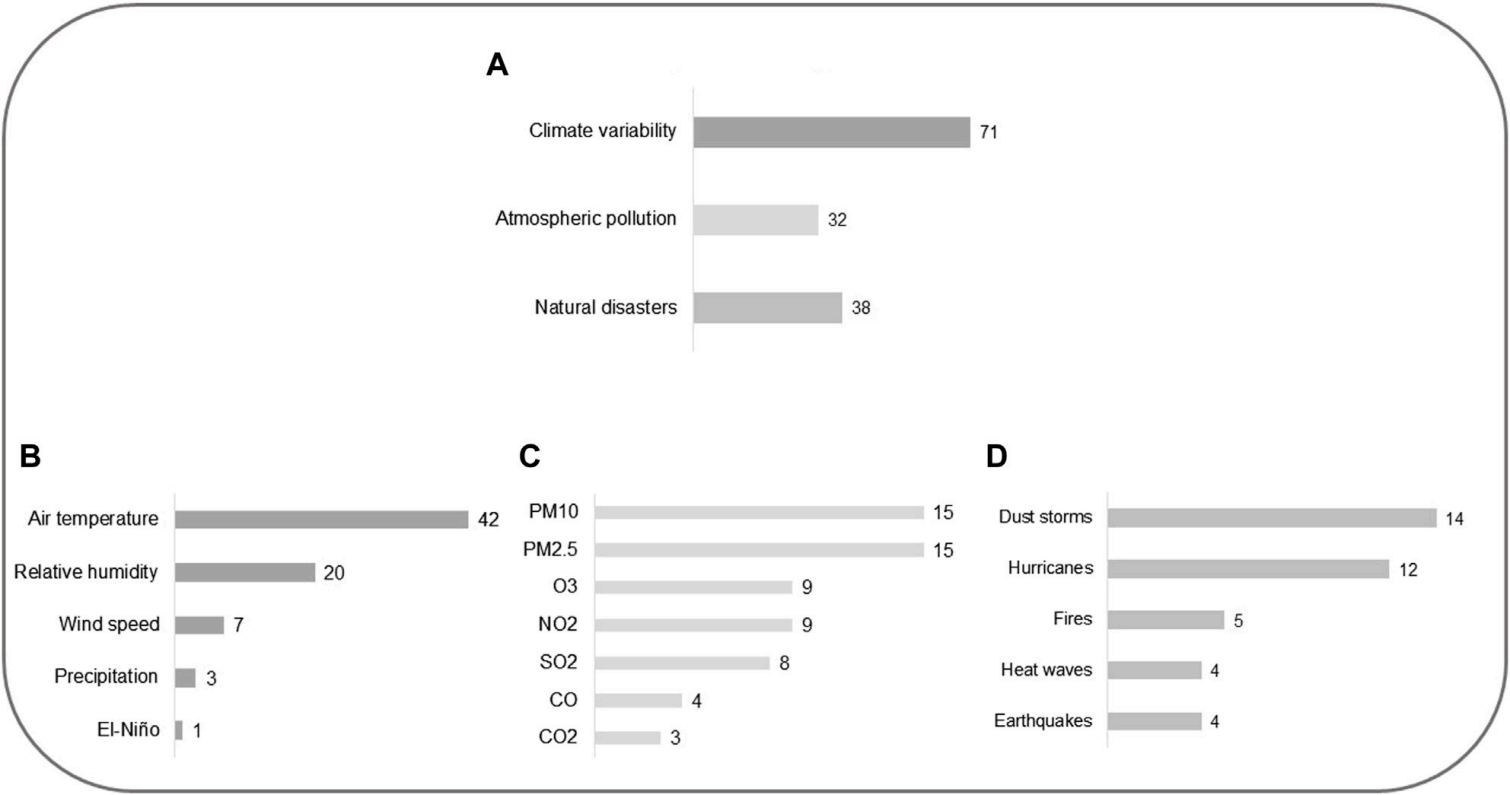
Central thematic categories emerging from data analysis: climate variability, atmospheric pollution, and natural disasters (2023, Brazil).

In this research, three central thematic categories were developed from data analysis: climate variability, atmospheric pollution, and natural disasters (Figure 3 and Supplementary Appendix Tables S8–S11). These categories are interconnected in influencing the development of cardiovascular diseases and the exacerbation of diabetes mellitus in dry regions, as we will discuss below.

The variation of climate variables (precipitation, air temperature, air humidity, and wind speed) and El Niño were understood as part of the climate variability category (n = 71). Within this category, studies were distributed as follows: air temperature (n = 42), relative air humidity (n = 20), wind speed (n = 7), precipitation (n = 3), and El Niño (n = 1). Additionally, 22 studies mentioned climate variability, but did not specify the variable or phenomenon itself. In the atmospheric pollution category (n = 32), pollution by particulate matter (PM10, PM2.5), O_3_, NO_2_, SO_2_, CO, and CO_2_ were included. Studies focused on each pollutant were distributed as follows: PM10 (n = 15), PM2.5 (n = 15), O_3_ (n = 9), NO_2_ (n = 9), SO_2_ (n = 8), CO (n = 4), and CO_2_ (n = 3). Extreme natural events, whether climatic or not, were categorized as natural disasters (earthquake, hurricane, fire, heatwaves, and dust storms). Studies in the natural disasters category (n = 38) were distributed as follows: earthquake (n = 4), hurricane (n = 12), dust storms (n = 14), fire (n = 5), and heatwaves (n = 4). Some studies mentioned more than one climate variable, pollutant, and/or natural disaster, as detailed in Supplementary Appendix Tables S9–S11.

In studies focusing on the influence of climatic components, it was observed that high precipitation was associated with more cases of CVD [37]. Air temperature significantly above or below historical averages is linked to increased CVD mortality [38]. The extreme heat characteristic of drought episodes is considered an important risk factor related to CVD [39]. Heatwaves are a causative factor of stress and exhaustion, posing risks to individuals with CVD [40]. Conversely, extreme decreases in temperature compared to historical averages can also lead to cardiovascular problems such as AMI and stroke [41].

The rise in air temperature, decrease in relative air humidity, and increase in wind speed can exacerbate the harmful effects of air pollutants on cardiovascular health. Increased temperature influences the concentration of air pollutants such as SO_2_, O_3_, NO_2_, PM10, and PM2.5 [42].

Dust storms, characterized by strong winds, elevate the concentration of air pollutants of natural or anthropogenic origin, such as SO_2_, O_3_, NO_2_, PM10, and PM2.5 [41–43]. These pollutants, along with CO_2_, have been associated with the formation of atherosclerotic plaques, leading to blood vessel obstruction [44].

Studies indicate that days with low relative air humidity have higher levels of air pollutants [45]. Additionally, dust storms also occur during periods of low relative air humidity, leading to increased hospital admissions for CVD [46].

The influence of the El Niño phenomenon on cardiovascular diseases is noteworthy. Meteorological droughts caused by El Niño in certain regions of the planet can intensify the risk of forest fires. These fires, in turn, emit gases and increase the concentration of air pollutants. Thus, a higher number of deaths and hospital admissions due to stroke are associated with El Niño occurrences [47].

**FIGURE 4:**
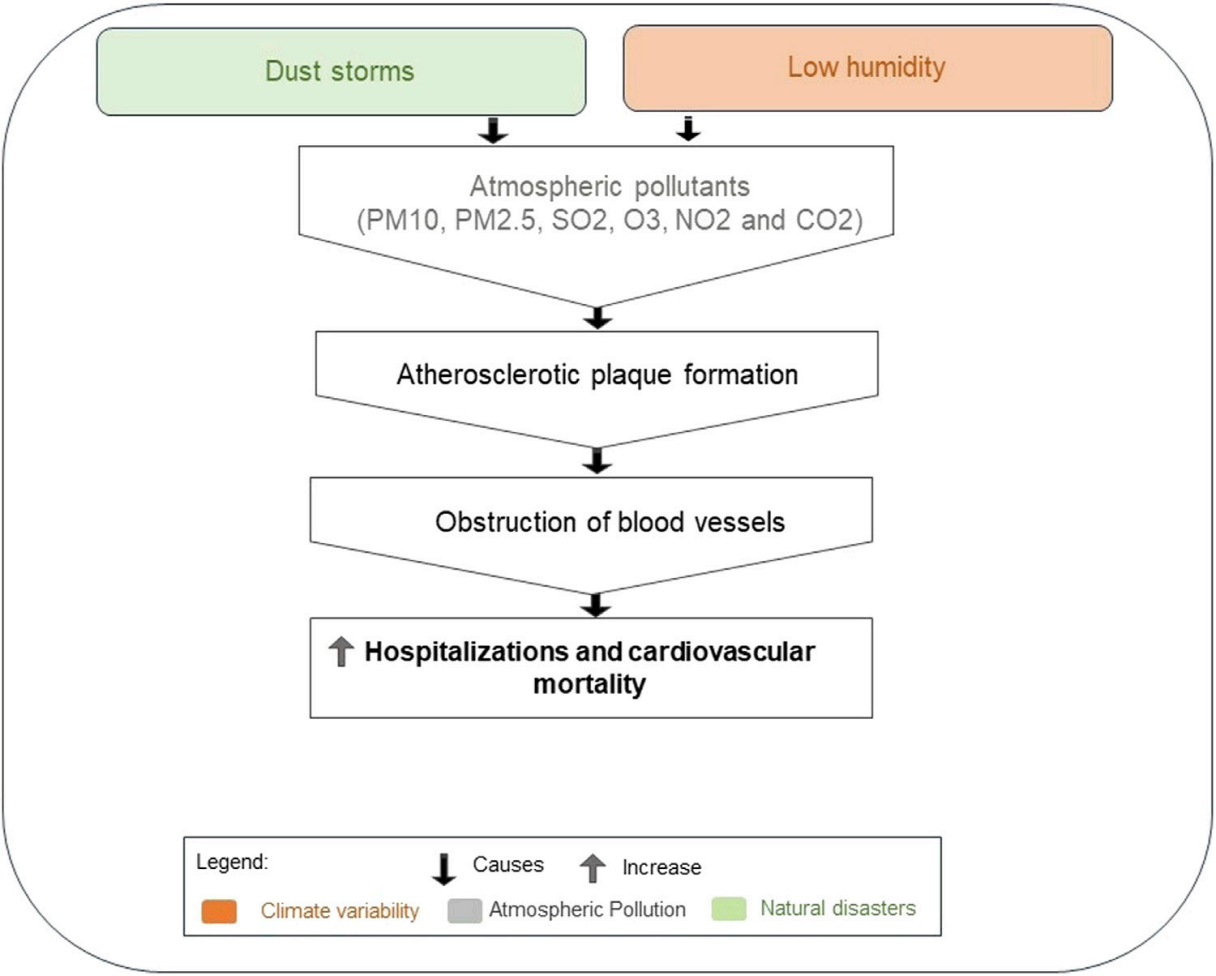
Effect of climate variables, atmospheric pollution, and natural disasters on increased hospitalizations and cardiovascular mortality (2023, Brazil).

**FIGURE 5:**
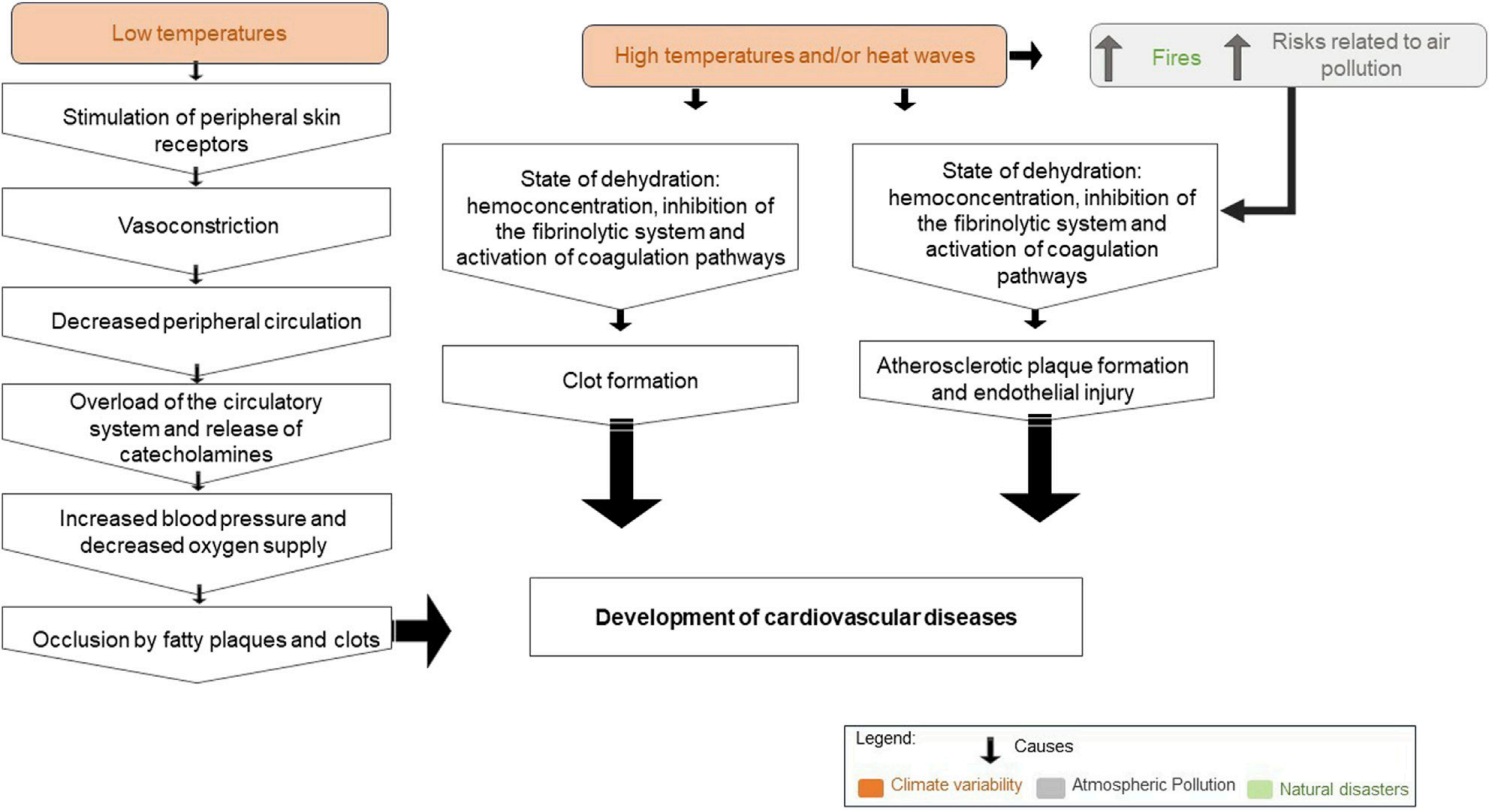
Effect of climate variables, atmospheric pollution, and natural disasters on the development of cardiovascular diseases (2023, Brazil).

The main effects mentioned in studies relating climatic variables, atmospheric pollution, and natural disasters to the development of CVD are summarized in Figures 4, 5.

Natural disasters such as heatwaves, dust storms, and wildfires are risk factors and have a direct impact on the development of cardiovascular diseases, as discussed above. However, there are disasters like earthquakes and hurricanes that have an indirect impact (Supplementary Appendix Table S11). Exposure to these disasters (earthquakes and hurricanes) contributes to exacerbating the effect of other risk factors in people with CVD and DM, such as: stress (n = 3), inadequate diet (n = 2), socioeconomic vulnerability (n = 4); medication unavailability, non-adherence, and treatment interruption (n = 3); increased smoking (n = 3); unstable blood sugar levels (n = 2); increased alcohol consumption (n = 2); and difficulty accessing healthcare services (n = 2), as shown in Supplementary Appendix Figure S3. These risk factors are further explored in Supplementary Appendix Table S12 and Supplementary Appendix S2.

Due to disasters such as earthquakes or hurricanes, individuals residing in affected areas need to relocate to refuge locations. In this process, many people are unable to bring the necessary medications for the treatment of chronic diseases such as diabetes mellitus and hypertension, discontinuing treatment [48], which can lead to alterations in blood glucose and/or blood pressure levels. Additionally, the food offered in shelters is often restricted and inadequate for people with CVD and DM, posing another risk factor for individuals to experience episodes of hyperglycemia, severe hypoglycemia, or hypertensive crises [49].

## Discussion

This scoping review allowed us to map the influence of variations in climatic variables (precipitation, air temperature, air humidity, and wind speed), El Niño, atmospheric pollution (PM10, PM2.5, CO_2_, O_3_, SO_2_, and NO_2_), and natural disasters (earthquake, hurricane, fire, heatwaves, and dust storms) on cardiovascular diseases and diabetes mellitus in dry regions. The most frequent diseases addressed in the studies were AMI and stroke. Exposure to extreme temperatures and atmospheric pollutants were the main risk factors with direct impacts on CVD, while exposure to natural disasters was an important risk factor for amplifying other known risk factors (inadequate diet, alcohol consumption, smoking, and stress), thus considered a risk factor with indirect impact on CVD and DM.

When analyzing the category of climatic variability, one of the studies included in this review related increased precipitation to increased CVD [37]. Similarly, an ecological study conducted in China identified an increase in hospitalizations due to ischemic stroke associated with increased precipitation, especially in men and older adults (>65 years) [50]. Other researchers observed in a cohort study a higher risk of incidence of stroke (ischemic and hemorrhagic) when rainfall was higher [51].

Additionally, the risk of stroke may increase when precipitation is associated with other climatic variables such as air humidity, as in environments with very high precipitation and higher humidity, the body’s decreased ability to sweat and the blockage of chloride and sodium discharge can lead to stroke [52]. On the other hand, low precipitation is observed in dry periods [53] and has been considered a risk factor for CVD, such as stroke [39]. In summary, it is known that extremes of high or low precipitation can increase the risk of stroke, even though there is no consensus in the literature on the type of stroke.

Regarding temperature variations, it was observed that, isolated or associated with other climatic variables, it was the one that most influenced the development or worsening of CVD. In the studies, we observed that the increase or decrease in temperature reference values was associated with CVD in the region where each study was conducted, with the association between increased temperature and heatwave being more frequent with the worsening or development of any cardiovascular disease [40].

In China, on days when heatwaves were present, there was an increased risk of various CVDs: chronic ischemic heart disease (32.2%), acute (27.3%), cerebrovascular disease (26.7%), and myocardial infarction (25.2%) [54]. According to the literature, the main pathophysiological mechanisms that can explain the relationship between increased temperature and CVD are dehydration, hemoconcentration, hypercoagulable state, and electrolyte disturbances [55]. Moreover, the formation of clots from hypercoagulation can result in acute coronary events or stroke [26]. We found in this research that heat stress and exhaustion resulting from heatwaves generated by the rise in temperature can pose risks for people with CVD [40]. According to Coates et al. [56], heatwaves already cause more deaths than all other natural disasters combined.

Similar to our research, other studies have also linked decreases in temperature to an increased incidence of myocardial infarction [57] and stroke [58]. The impact of low temperatures on these cardiovascular events may be connected to vasoconstriction—a natural response of the human body to cold. Vasoconstriction aims to reduce heat loss by narrowing blood vessels. However, this narrowing can raise the risk of myocardial infarction by reducing peripheral blood circulation. Additionally, it may promote the occlusion of arteries due to fat plaques and blood clots. Furthermore, cold temperatures can lead to elevated blood pressure and decreased oxygen supply [59, 60], further contributing to cardiovascular risks.

In addition to the isolated influence of climate variability on CVD, some of the studies included in this review also highlighted the effects of these climatic variables on the concentration of air pollutants in the development of CVD. A higher concentration of air pollutants was observed during periods of low air humidity in dry seasons, contributing to increased cardiovascular hospitalizations and mortality [46]. Also, low humidity, combined with the high temperatures typical of dry seasons, provides favorable conditions for dust storms [61] and fires [62]. During dust storms, hot air masses can intensively concentrate local pollutants [63], besides favoring the transport and condensation of gaseous pollutants such as SO_2_ [64].

Just like dust storms, temperature variation, and heatwaves influence the increase in concentration of air pollutants harmful to cardiovascular health, such as greenhouse gases [42], which can accentuate CVD mortality. Particulate matter along with CO_2_ has been associated with the formation of atherosclerotic plaques, stemming from blood hypercoagulability due to endothelial dysfunction [44], exposing individuals to the risk of myocardial infarction and heart failure [39, 41].

The pathophysiological mechanisms by which exposure to air pollution negatively affects cardiovascular diseases are not yet fully understood; however, studies have highlighted possible explanations such as systemic inflammatory response [28], oxidative stress [65], genetic and epigenetic factors, impact on the central nervous system and autonomic dysfunction, as well as prothrombotic response, vascular dysfunction, and remodeling [66].

The last category presented in this study was that of natural disasters, being the only one where studies directed at people with DM were identified. Natural disasters, such as hurricanes [49] and earthquakes [48, 67], can be risk factors that cause indirect impacts, as they lead to the development or intensification of other risk factors, such as behavioral factors (tobacco and alcohol use, sedentary lifestyle, among others) and/or hypertension, which can trigger and/or exacerbate cardiovascular problems and diabetes mellitus.

After a disaster situation, a person may experience a hypertensive crisis due to sudden stress. Also, a gradual increase in blood pressure values is common due to the immediate and unplanned need for lifestyle changes, such as inadequate meals leading to weight gain, and the possibility of increased alcohol consumption. This finding is supported by a study conducted in the city of Fukushima, Japan, on the evacuation experience of individuals after a hurricane, which found an increase in blood pressure among these individuals [68]. After a disaster, in addition to changes in blood pressure values, alterations in HbA1c levels in people with DM are common, increasing the risk of hospitalizations [69].

Dietary habits may change after a natural disaster, for example, individuals in temporary shelters are restricted to the meals provided there [49, 67, 70]. Another influencing factor may be the distance from supermarkets and fast-food outlets when individuals are relocated to areas farther away from these establishments [71], reducing their opportunities to obtain the foods that were part of their diet.

Changes in dietary patterns can also occur due to mental illness, such as symptoms of post-traumatic stress disorder (PTSD) and depression [72], thereby increasing the risk of CVD and DM. The occurrence of late-onset post-traumatic stress disorder among survivors of natural disasters may be associated with impaired self-control [73]. A study conducted in Japan after the major earthquake and tsunami in 2011 suggested that the increased risks of cardiometabolic diseases may result from changes in diet associated with PTSD and depression. There was an association between reduced healthy eating patterns and depressive and/or PTSD symptoms, leading to increased unhealthy eating habits such as higher consumption of processed foods rich in carbohydrates and proteins, reduced intake of fruits and vegetables, and increased alcohol consumption, especially among males [74].

The likelihood of using toxic substances among survivors of natural disasters was also identified in our study. The main concerns of survivors were directed towards potential family or material losses, which can lead individuals to high levels of physical and emotional stress [49, 67, 75], favoring increased cigarette smoking [38, 70, 75] or alcohol consumption [38]. This finding may be associated with psychological distress resulting from decreased self-efficacy perception in coping, leading to increased substance use post-natural disaster [76]. Alcohol use as a temporary relief for stress is also common [77].

Other conditions reported in studies and related to increased hospitalization or mortality from CVD or DM in individuals affected by natural disasters include interruption of routine healthcare services [78] and lack of access to medications and treatments [48, 49, 67, 79] due to increased difficulty in accessing services [70]. All of this may be related to difficulty in self-managing some chronic diseases post-natural disaster [80].

The destruction of facilities, infrastructure, and significant changes in living conditions caused by natural disasters can increase difficulty, especially in older people, in maintaining healthy practices [77]. In addition to causing the destruction of health facilities, natural disasters can also reduce the number of professionals and interfere with the quality and effectiveness of health services provided [81, 82]. Furthermore, in circumstances of natural disaster, health actions often focus on patients with acute conditions such as trauma or infectious diseases, even though inadequate control of chronic diseases is considered a major threat to people’s health [82].

The most vulnerable groups identified in this review are similar to other research, with a focus on older adults, women, and children, who are most affected by climate influence [83]. This association may occur due to the greater susceptibility of older adults to retain more heat when exposed to hot and dry conditions than younger individuals [84]. The presence of comorbidities, such as hypertension, may further increase this vulnerability of older adults to heat exposure [85].

Women were also indicated in the studies as one of the vulnerable groups. This result may be associated with the progression of atherosclerotic disease. In women, the progression of atheroma plaques is slower but more severe [86] and may be exacerbated by climate change. Children were also considered vulnerable in the analyzed studies. This relationship may be related to the physiological development of children being different from adults. Furthermore, it is worth noting that children are exposed to long-term extreme weather events [87], as recent studies indicate that children born in 2020 will be more exposed to heatwaves (7 times more) and wildfires (2 times more) than previous generations in recent decades [88].

Exposure to climatic variability, pollution, and natural disasters in drylands is linked to cardiovascular diseases. Natural disasters also indirectly worsen diabetes mellitus. Vulnerability is particularly pronounced among older adults, women, and children. These factors often interact rather than act independently, highlighting the importance of nations adhering to Paris Agreement targets to decrease air pollutants and enhance resilience against disasters. Further research is imperative for tailored health interventions, necessitating interdisciplinary teams to alleviate the health impacts of climate, pollution, and disasters on these conditions.

### Study Limitations

The study has limitations due to variability in population size and composition across the studies analyzed. Moreover, factors like public policies (such as early warning systems or green spaces) and socioeconomic status (income) could influence the results but were not addressed in this study.
