# Supplementary material for: The Influence of Climate, Atmospheric Pollution, and Natural Disasters on Cardiovascular Diseases and Diabetes Mellitus in Drylands: A Scoping Review
Source: Public Health Rev. 2024 Aug 8;45:1607300. doi: 10.3389/phrs.2024.1607300 (PMC11338784; doi:10.3389/phrs.2024.1607300)
Supplement: Supplementary file 1 [file DataSheet2.docx]

**Supplementary appendix 2**

**Table S13: Synthesis of studies on the relationship between climate, atmospheric pollution, natural disasters, and cardiovascular diseases in drylands**

| **ID** | **Database** | **Authors** | **Title** | **Year** | **Country** | **Study design** | **Objectives** | **Results** |
| --- | --- | --- | --- | --- | --- | --- | --- | --- |
| 1 | EMBASE | KR Van Daalen et al. | The 2022 Europe report of the Lancet Countdown on health and climate change: towards a climate resilient future | 2022 | Other countries | Report | To track progress on health and climate change in five areas: climate change impacts, exposures, and vulnerabilities; adaptation, planning, and resilience for health; mitigation actions and health co-benefits; economics and finance; and politics and governance. | Wildfire smoke is associated with increased mortality, morbidity, and hospital admissions and exacerbates respiratory and cardiovascular conditions; heat exposure has increased by 57% between the first and second decade of the 21st century |
| 2 | SCOPUS | M. Dahmardeh | Assessment of drought damage of Hamoun wetland on health condition of inhabitants of Sistan region | 2016 | Iran | Quantitative | To assess the adverse effects of dust particles resulting from Hamoun wetland on human health in Sistan in 2014. The computerized model Air Q2.2.3 22 was used. | Each 10 μg/m3 pollution concentration can increase the rate of natural death by 0.5%, whereas this rate for the cardiovascular and respiratory patients is 1 and 2%, respectively. However, due to complete dryness of the wetland, 920,531 μg/m3 pollution concentrations can increase the rate of natural death by 12.61%, whereas this rate is 19.69 and 50.44% for the cardiovascular and respiratory patients. |
| 3 | EBSCO | J. L. C. Crooks, Wayne E.; Percy, Madelyn S.; Reyes, Jeanette; Neas, Lucas M.; Hilborn, Elizabeth D. | The association between dust storms and daily non-accidental mortality in the United States, 1993-2005 | 2016 | United States | Quantitative | To examine the association between dust storms and county-level non-accidental mortality in the United States from 1993 through 2005. | For the United States as a whole, total non-accidental mortality increased by 7.4% (95% CI: 1.6, 13.5; p = 0.011) and 6.7% (95% CI: 1.1, 12.6; p = 0.018) at 2- and 3-day lags, respectively, and by an average of 2.7% (95% CI: 0.4, 5.1; p = 0.023) over lags 0–5 compared with referent days. Significant associations with non-accidental mortality were estimated for California (lag 2 and 0–5 days) and Arizona (lag 3), for cardiovascular mortality in the United States (lag 2) and Arizona (lag 3), and for other non-accidental mortality in California (lags 1–3 and 0–5). |
| 4 | EBSCO | BMR Longo, A.; Green, J.B. | Cardiorespiratory health effects associated with sulphurous volcanic air pollution | 2008 | United States | Environmental–epidemiological cross-sectional study | To investigate cardiorespiratory health effects associated with chronic exposure to volcanogenic sulfur dioxide (SO2) and fine sulphate particle (p0.3 mm) air pollution emitted from Kilauea Volcano, Hawaii. | There were statistically significant positive associations between chronic exposure and increased prevalence of cough, phlegm, rhinorrhoea, sore/dry throat, sinus congestion, wheezing, eye irritation and bronchitis. The magnitude of the associations differed according to SO2 and fine sulphate particulate exposure. Group analyzes found no differences in pulse rate or BP; however, significantly faster mean pulse rates were detected in exposed non-medicated, non-smoking participants with BMI o25, and in participants aged X65 years. Higher mean systolic BP was found in exposed participants with BMI o25. |
| 5 | EBSCO | C.-CN Chan, Huey-Ching | A case-crossover analysis of Asian dust storms and mortality in the downwind areas using 14-year data in Taipei | 2011 | Taiwan | Quantitative | To assess the association between ADS and daily mortality of residents in Taipei metropolis, Taiwan, by applying a case-crossover design to a longer observational period of 14 years between 1994 and 2007. | Age-stratified analysis showed that total non-accidental and cardiovascular deaths were significantly raised only for people above 65 years old during Asian dust days, with OR= 1.025 (95% CI 1.006–1.044) and OR= 1.045 (95% CI 1.011– 1.081), respectively, with no statistically significant effects for those aged below 65. We also found marginally statistically significant (pb0.10) associations between ADS and cardiovascular deaths of all ages on dust storm days, and total non-accidental deaths for residents aged below 65 on the next day after ADS. In addition, there were no statistically significant effects of ADS on excess respiratory deaths in Taipei. |
| 6 | PUBMED/MEDLINE | Raul Cruz-Cano; Erin L. Mead | Causes of excess deaths in Puerto Rico after Hurricane Maria: a time-series estimation | 2019 | Puerto Rico | Time-series study | To determine the number and causes of excess deaths in Puerto Rico after Hurricane Maria made landfall on September 20, 2017. | Expected mortality rates were 8.08 per 1000 people in September 2017 and 8.24 per 1000 in October 2017. Excess deaths were higher among men than women (632 and 579 deaths, respectively) and occurred only among people aged 60 years or older (1038 deaths) . Most excess deaths were the result of heart disease (253 deaths), “other” causes (204 deaths), diabetes (195 deaths), Alzheimer's disease (122 deaths), and septicemia (81 deaths). |
| 7 | EBSCO | K.-SH Huang, Ding-Xiu; Huang, De-Jia; Tao, Qian-Lan; Deng, Xiao-Jian; Zhang, Biao; Mai, Gang; Guha-Sapir, Debarati | Changes in ischemic heart disease mortality at the global level and their associations with natural disasters: A 28-year ecological trend study in 193 countries | 2021 | Other countries | Ecological analysis | To investigate the hypothesis that natural disasters—as an environmental factor—are associated with Ischemic Heart Disease (IHD) mortality and Years of Life Lost (YLL) rates. | Significant changes were found in the IHD mortality and YLL rates and the occurrence of disasters between the two equal periods (1990 to 2003 and 2004 to 2017) (p<0.001). The bivariate Pearson correlation test revealed that the trend in the occurrence of natural disasters was positively correlated with trends in the IHD mortality and YLL rates among females |
| 8 | EBSCO | K.-SH Huang, Ding-Xiu; Tao, Qianlan; Wang, Yan-Yan; Yang, Yong-Qiang; Zhang, Biao; Mai, Gang; Guha-Sapir, Debarati | Changes in the incidence and prevalence of ischemic stroke and associations with natural disasters: an ecological study in 193 countries | 2022 | Other countries | Ecological analysis | To determine the associations between natural disasters and the incidence and prevalence of ischemic stroke at the global level. | Changes in the incidence and prevalence of ischemic stroke, as well as disaster occurrence, varied among the different regions over the past 28 years (p < 0.001). Multiple linear regression revealed an independent and positive association between disaster occurrence and the incidence of ischemic stroke in males, females and both sexes combined (standardized coefficients= 0.515, 0.470 and 0.483, p < 0.001); similar associations were found for the prevalence of ischemic stroke (standardized coefficients= 0.471, 0.417 and 0.438, p < 0.001). The incidence and prevalence of ischemic stroke changed significantly at the global level and were independently associated with natural disasters. |
| 9 | EBSCO | ACA Miller, B. | Chronic diseases and natural hazards: impact of disasters on diabetic, renal, and cardiac patients | 2008 | Other countries | Literature review | To assess the burden of chronic renal failure, diabetes, and cardiovascular disease during disasters due to natural hazards, identify impediments to care, and propose solutions to improve the disaster preparation and management of CDE. | Diabetic and cardiac patients are probably better served by improving the preparation and response of established relief agencies. Patients must be educated on disaster preparation, which may range from the large scale to local practices and drills. It is urged that patients wear some form of medical alert identification, and that they carry essential medical information on their person such as the patient databases provided. Patients must be familiar |
| 10 | PUBMED/MEDLINE | HA Khraishah, B.; Ostergard, R.L., Jr.; AlAshqar, A.; Albaghdadi, M.; Vellanki, N.; Chowdhury, MM; Al-Kindi, SG; Zanobetti, A.; Gasparrini, A.; Rajagopalan, S. | Climate change and cardiovascular disease: implications for global health | 2022 | Other countries | Literature review | To explore the effect of air pollution, extreme temperatures and other extreme weather events on CVD | The effect of climate change on cardiovascular and overall health is a multifaceted problem that needs to be addressed at various levels. In the most favorable outlook for climate change in the future, alterations in temperature, air quality and extreme weather will still result in quantifiable and avoidable cardiovascular events. |
| 11 | SCOPUS | JP Besancenot | Climate change and health | 2015 | Other countries | Review | To present a review of current knowledge and hypotheses on the subject, focusing on key points for which significant advances have been made since 2012. | Direct observed and projected effects on health include primarily thermal stresses due to the increased frequency, intensity, or persistence of heat waves, which can trigger heat exhaustion and heatstroke as well as cardiovascular and respiratory diseases. Any decrease in cold-related deaths cannot outweigh this increase in those related to heat. Other extreme weather events, such as long drought periods, severe storms, heavy precipitation, and tropical cyclones, can also have adverse outcomes and cause injury and death. Among the indirect effects of climate change are the alteration of ecosystems, disruption of food production and of safe water supplies, the worsening of air quality, and all of their consequences for infectious (especially vectorborne) diseases. |
| 12 | EBSCO | MB-F. Akpinar-Elci, Brenda; Bayram, Hasan; Al-Taiar, Abdullah | Climate change, dust storms, vulnerable populations, and health in the Middle East: a review | 2021 | Other countries | Systematic review | To identify and assess what is currently understood about the health impacts of dust storms in the Middle East. | Dust storm events and dust were associated with increased risk for hospital admissions for both respiratory and cardiovascular events, reductions in lung function and capacity, irregularities in blood coagulation and increases in allergic inflammation, as well as increased risk for diseases associated with exposures to airborne humans pathogens. |
| 13 | SCIELO | AMMV Rodríguez Bertheau, Miriam; Martínez Rodríguez, Ileana; Fundora Hernández, Hermes; Guzmán Armenteros, Tania | Technological development, environmental impact and health | 2011 | Other countries | Qualitative | To address the current problems existing between the technological development and its influence on the environment and human health, as well as the different points of view with which a solution to this difficult crossroads. | Quantities of CO2 were released and other greenhouse gases that have caused an estimated excess mortality of 70,000 deaths in Europe, and aggravate cardiovascular and respiratory diseases with 1.2 million deaths each year in urban areas. |
| 14 | SCOPUS | PM Kinay, AP; Villanueva, EV; Morrissey, K.; Staddon, P.L. | Direct and indirect health impacts of climate change on the vulnerable elderly population in East China | 2019 | China | Literature review | To review and summarize the latest scientific advances in understanding the likely future health impacts of climate change. | The review highlights the broad range of health risks linked to climate change and identifies where more research effort is needed. In particular, more quantitative and epidemiological studies are required to better understand the likely impact of climate change on the health of the growing population of older adults. |
| 15 | PUBMED/MEDLINE | Jesse D Berman, Keita Ebisu, Roger D Peng, Francesca Dominici, Michelle L Bell | Drought and the risk of hospital admissions and mortality in older adults in western USA from 2000 to 2013: a retrospective study | 2017 | United States | Ecological study | To estimate the risks of hospitalization and mortality related to cardiovascular and respiratory diseases associated with drought conditions for older adults of the western US. | Cardiovascular admissions did not differ significantly during periods of drought or worsening drought. In counties where drought occurred less frequently, we found that cardiovascular disease and mortality risks increased during worsening drought conditions. Counties that experience fewer drought events have a higher risk of cardiovascular disease mortality and hospitalizations (0.72% increase). |
| 16 | SCOPUS | P ACHAKULWISUT, LJ MICKLEY AND SC ANENBERG | Drought-sensitivity of fine dust in the US Southwest: Implications for air quality and public health under future climate change | 2018 | United States | Ecological study | To investigate the current sensitivity of fine dust levels in the US Southwest to regional drought conditions and use the observed relationships to assess future changes in fine dust levels and associated health impacts under climate change. | The authors demonstrated that the 2000–2015 interannual variability of monthly mean fine dust concentrations across the southwestern United States is influenced by drought conditions in local and surrounding areas. Considering future population and baseline incidence rates, these increases in fine dust could lead to 860 (300%, RCP8.5) excess hospital admissions due to cardiovascular and respiratory disease each year for adults aged ≥65 years, compared to the present day |
| 17 | EBSCO | HO Aghababaeian, Abbas; Ardalan, Ali; Asgary, Ali; Akbary, Mehry; Yekaninejad, Mir Saeed; Sharafkhani, Rahim; Stephens, Carolyn | Effect of dust storms on non-accidental, cardiovascular, and respiratory mortality: a case of Dezful City in Iran | 2021 | Iran | Quantitative | To assess the association between dust storms and daily non-accidental, cardiovascular, and respiratory mortality in Dezful City (Khuzestan Province, Iran) during 2014 to 2019. | During the study period, 15223 deaths were recorded, and 139 dust storms occurred in Dezful city. In addition, there was statistically significant excess risk of mortality due to dust storms in Dezful City (mortality in the group under 15 years of age, lag4: 34.17% and 15-64years of age groups, lag5: 32.19%, lag6: 3.28 %), also dust storms had statistically significant effects on respiratory mortality (lag6: 5.49%). |
| 18 | EBSCO | C.-YC Yang, Yong-Shing; Chiu, Hui-Fen; Goggins, William B. | Effects of Asian dust storm events on daily stroke admissions in Taipei, Taiwan | 2005 | Taiwan | Quantitative | To assess the possible associations of ADS on the hospital stroke admissions of residents in Taipei, Taiwan, during the period from 1996 to 2001. | The study results indicated a statistically significant association between ADS events and daily primary intracerebral hemorrhagic stroke admissions 3 days after the event (relative risk of 1.15; 95% CI, 1.01–10.10). We also found a positive but nonsignificant association between ADS events and ischemic stroke admissions 3 days following the dust storms. This was mainly due to PM10. |
| 19 | PUBMED/MEDLINE | PA Achakulwisut, SC; Neumann, J.E.; Penn, SL; Weiss, N.; Crimmins, A.; Fann, N.; Martinich, J.; Roman, H.; Mickley, L.J. | Effects of increasing aridity on ambient dust and public health in the U.A. Southwest under climate change | 2019 | United States | Ecological study | To quantify the resulting impacts on ambient dust levels and public health using methods consistent with the Environmental Protection Agency's Climate Change Impacts and Risk Analysis framework. | Findings suggest that airborne levels of soil-derived components of PM may increase in the southwestern US due to increased aridity, especially under RCP8.5. In 2090, according to RCP8.5 for the reference period, most premature mortality attributable to fine dust is due to cardiopulmonary deaths and has the highest incidence rates for adults aged 75 years and older. Estimated A2018 through 2095, all-cause mortality could grow by 750 deaths per year (130%) and hospital admissions related to cardiovascular problems (fewer myocardial infarctions) and respiratory diseases could grow by 860 admissions per year (300%). |
| 20 | EBSCO | S. W. Weinrich, M.; Hardin, S.; Gleaton, J.; Pesut, DJ; Garrison, C. | Effects of psychological distress on blood pressure in adolescents | 2000 | Columbia | Cross-sectional study | To measure relationships among blood pressure and measures of psychological distress, family structure, and economic status in a sample of adolescents exposed to Hurricane Hugo | Data analysis revealed 5% of the 1079 adolescents were hypertensive. Multiple regression analyzes revealed the following predictors of higher diastolic blood pressure: African-American race, recipient of subsidized lunch, exposure to Hurricane Hugo, and higher anger- in scores in evils. The effects of a catastrophic event such as a hurricane on blood pressure and the effects of introjected anger have implications for both health care consumers and providers. |
| 21 | PUBMED/MEDLINE | MED Marlier, RS; Voulgarakis, A.; Kinney, PL; Randerson, J.T.; Shindell, D.T.; Chen, Y.; Faluvegi, G. | El Niño and health risks from landscape fire emissions in Southeast Asia | 2013 | Other countries | Quantitative | To quantify health effects from fire emissions in southeast Asia from 1997 to 2006 | During strong El Niño years, fires contribute up to 200 μg m−3 and 50 ppb in annual average fine particulate matter (PM2.5) and ozone surface concentrations near fire sources, respectively. This corresponds to a fire contribution of 200 additional days per year that exceeds the World Health Organization 50 μg m−3 24-hr PM2.5 interim target4 and an estimated 10,800 (6,800–14,300)-person (∼2%) annual increase in regional adult cardiovascular mortality. |
| 22 | PUBMED/MEDLINE | CLT Hua, Kali S.; Peterson, Lindsay J.; Hyer, Kathryn; Dosa, David M. | Emergency department use among assisted living residents after Hurricane Irma | 2021 | United States | Retrospective cohort study | To examine whether emergency department finger use rates for injuries and other medical reasons increased after Hurricane Irma in 2017 among Florida AL residents. | Heart failure rates were higher in 2017 (After the hurricane) compared to 2016 (Before the hurricane) |
| 23 | PUBMED/MEDLINE | Weilnhammer V, Schmid J, Mittermeier I, Schreiber F, Jiang L, Pastuhovic V, Herr C, Heinze S | Extreme weather events in europe and their health consequences – A systematic review | 2021 | Other countries | Systematic review | To assess the available evidence on associations between Weather events in Europe and their impact on health. | Most studies suggested that extreme heat or cold events lead to increased overall and cause-specific mortality. The effects of droughts are unclear due to their simultaneous occurrence with heat waves, forest fires or air pollution. Forest fires increase overall mortality and cardiovascular mortality within the European population. |
| 24 | EBSCO | Aghababaeian H, Ostadtaghizadeh A, Ardalan A, Asgary A, Akbary M, Yekaninejad MS, Stephens C. | Global health impacts of dust storms: a systematic review | 2021 | Taiwan, China, Spain and Iran | Systematic review | To examine the health impacts of dust storms around the world to provide an overview of this issue | Most studies show that dust storms increase the risk of cardiovascular problems, the number of cardiovascular emergency medical dispatches, cardiovascular visits, the number of cardiovascular symptoms among patients referring to the hospital, cardiovascular admissions or hospitalizations, and deaths due to cardiovascular disease. |
| 25 | SCOPUS | RJB Rocque, C.; Ndjaboue, R.; Cameron, L.; Poirier-Bergeron, L.; Poulin-Rheault, RA; Fallon, C.; Tricco, AC; Witteman, H.O. | Health effects of climate change: an overview of systematic reviews | 2021 | Other countries | Review | To develop a systematic synthesis of systematic reviews of health impacts of climate change, by synthesizing studies' characteristics, climate impacts, health outcomes and key findings | Temperature and humidity are the most studied variables and report the most consistent associations with infectious and respiratory, cardiovascular, and neurological diseases outcomes.associations are explored less often, but these studies suggest an association between drought and respiratory and cardiovascular problems (probably through air quality ). Interdependence can be direct (eg, impact of heat on dehydration and exhaustion) or indirect (eg, through behavioral change due to heat). respiratory, cardiovascular and neurological. |
| 26 | EBSCO | Bell SA, Donnelly JP, Li W, Davis MA. | Hospitalizations for chronic conditions following hurricanes among older adults: a self-controlled case series analysis | 2022 | United States | Case series analysis | To characterize hospitalizations among older adults by chronic condition after eight large-scale hurricanes in the United States. | Among all exposed older adults, hospitalizations within 30 days of each disaster increased for all three chronic conditions; diabetes (incidence rate ratio [IRR] = 1.06, 95% confidence interval [CI] 1.03, 1.10), COPD (IRR = 1.06, 95% CI 1.04, 1.08) and CHF (IRR = 1.19, 95% CI 1.17, 1.21. In the 30- to 60-day period, hospitalizations also increased for each chronic condition; diabetes (IRR = 1.06, 95% CI 1.03, 1.10), COPD (IRR = 1.12, 95% CI 1.10, 1.15) and CHF ( IRR = 1.32, 95% CI 1.30, 1.34) |
| 27 | EBSCO | WTS Cefalu, SR; Blonde, L.; Fonseca, V.; Cefalu, William T.; Smith, Steven R.; Blonde, Lawrence; Fonseca, Vivian | The hurricane Katrina aftermath and its impact on diabetes care | 2006 | United States | Review | To summarize observations on a disaster and advocate for the development of guidelines to aid health organizations, professionals, and individuals with chronic diseases in crafting a proactive "plan of action" for managing major disasters. | Abrupt change in dietary intake and/or composition with lack of diabetes medication led to a significant disruption in glycemic control, putting patients at risk of severe hyperglycemia and hypoglycemia.Financial loss, loss of personal belongings, and occasionally bereavement have led to severe depression, affecting many diabetes patients. |
| 28 | PUBMED/MEDLINE | MM Franchini, PM | Impact on human health of climate changes | 2015 | United States | Review narrative | To synthesize the most relevant current and potential aspects of climate change. | Extreme heat can trigger congestive heart failure attacks |
| 29 | BVS | BRM Sandoval D, Dennise; Reyes R, Tatiana; Oyarzún G, Manuel | Impact of air pollution by forest fires and the morbidity of the exposed population | 2021 | Chile | Literature review | To analyze the increase in con-air pollution during fires forests with vegetation similar to the national -excluding rice paddy fires-, and their deleterious effects on the health of the population exposed to the emanations of these fires | Exposure to wildfire smoke, tissue damage occurs, increased of prothrombotic mechanisms, increased blood pressure and changes in heart rhythm |
| 30 | EBSCO | Joshua Chen-Yuan Teng, Yun-Shan Chan, Yu-I Peng and Tsai-Ching Liu | Influence of Asian dust storms on daily acute myocardial infarction hospital admissions | 2015 | Taiwan | Time series models | To explore the relationship between Asian dust storm events (ADS) and hospital admissions for acute myocardial infarction (AMI) by application time series models. | There were 143,063 AMI admissions during 2000-2009. Hospitalizations for AMI increased significantly on the third day post-ADS, especially in the male population - ages 45-64 and over 74 years. |
| 31 | EBSCO | TA Quast, Ross; Sadhu, Archana R. | Long-term effects of disasters on seniors with diabetes: evidence from Hurricanes Katrina and Rita | 2019 | United States | Retrospective cohort analysis | To estimate the long-run mortality effects of Hurricanes Katrina and Rita on seniors with diabetes. | The affected subjects had a nearly 40% higher all-cause mortality risk in the 1st month after the storms, but the difference fell to <6% by the end of the full observation period. The mortality risks of heart disease and nephritis also exhibited the largest differences immediately following the storms. Among the affected subjects, the all-cause mortality risk was higher for those who moved to a different county, with an especially large difference among those who moved to an affected county. |
| 32 | EBSCO | Kathleen M. McDermott, BNurs, DipHSc, MPH;1 Ruth M. Hardstaff, MBBS, MD, MRCS, FRCS, FRACS;2 Sophie Alpen;1 David J. Read, MBBS, FRACS;1,2 Nicholas R. Coatsworth, MBBS , MIntPH, FRACP1 | Management of diabetic surgical patients in a deployed field hospital: a model for acute non-communicable disease care in disaster | 2017 | Australia | Surgical cohort | To detail the experience of paramedics during the 2013 Typhoon Haiyan disaster, with particular reference to the challenges of treating the diabetic in a surgical field hospital | The incidence of diabetes in the surgical cohort exceeded that of the population by a factor of four (30 of the 131 patients [22.9%] were diabetic). The steps to prepare for and treat diabetes in the field provide a useful model for the management of NCD in the deployed field hospital environment after a disaster. |
| 33 | PUBMED/MEDLINE | SMA Malik, H.; Khan, N. | Mapping vulnerability to climate change and its repercussions on human health in Pakistan | 2012 | Pakistan | Ecological study | To rank the agro-ecological zones in Pakistan according to their vulnerability to climate change and to identify the potential health repercussions of each manifestation of climate change in the context of Pakistan. | The findings show that Balochistan, with its high sensitivity and low adaptive capacity, is the most vulnerable region, followed by low-intensity Punjab (predominantly South Punjab) and Cotton/Wheat Sindh. Each region faces different health risks based on climate threats. Flooding increases the risk of diarrhea, gastroenteritis, skin and eye infections, acute respiratory infections, and malaria. Drought exposure poses risks of food insecurity, malnutrition, anemia, night blindness, and scurvy. Rising temperatures increase the risk of heat stroke, malaria, dengue, respiratory diseases, and cardiovascular diseases. |
| 34 | PUBMED/MEDLINE | MNM Peters, John C.; Katz, Morgan J.; Deandrade, Kevin B.; Quevedo, Henry C.; Tiwari, Sumit; Burchett, Andrew R.; Turnage, Thomas A.; Singh, Kanwar Y.; Fomunung, Edmond N.; Srivastav, Sudesh; Delafontaine, Patrice; Irimpen, Anand M. | Natural disasters and myocardial infarction: the six years after Hurricane Katrina | 2014 | United States | Retrospective cohort study | To determine the prolonged effect of Hurricane Katrina on the incidence and timing of acute myocardial infarction (AMI) in the city of New Orleans. | 1476 patients were analyzed for demographic data and clinical history. Higher rates of previously diagnosed coronary artery disease (P = 0.004), hyperlipidemia (P = 0.004). 05), psychiatric comorbidities (P = 0.01), smoking (P < 0.001) and lack of employment, in more post-earthquake groups. Majority were young people. |
| 35 | PUBMED/MEDLINE | FS Gohardehi, Hesam; Moslehi, Shandiz | Prevalence rate of diabetes and hypertension in disaster-exposed populations: a systematic review and meta-analysis | 2020 | Iran | Systematic review and meta-analysis | To examine the relationship between the prevalence of HTN and DM and the conditions created following natural or unnatural disasters that affect the human population. | This study found that the prevalence of HTN and DM was higher in survivors of disasters in comparison to the general population. |
| 36 | PUBMED/MEDLINE | T Gottlieb-Stroh; A Souares; T Bärnighausen; A Sié; SP Zabre; I Danquah | Seasonal and socio-demographic patterns of self-reporting major disease groups in north-west Burkina Faso: an analysis of the Nouna Health and Demographic Surveillance System (HDSS) data | 2021 | Burkina Faso | Quantitative | To identify the occurrence and co-occurrence of major disease groups in two different seasons, and the associations with socio-demographic factors among adults living in rural north-west Burkina Faso, mainly for the creation and prioritization of public health policies to be put in place . | In 2010 and 2011, 3,949 and 4,039 adults participated in surveys, respectively. Self-reported communicable diseases were more prevalent in the rainy season (20.7%) compared to the dry season (11.0%), while self-reported NCDs showed no significant seasonal difference. Injuries were reported by less than 1% each year. The overlap of communicable diseases and NCDs was rare (1.4% in 2010, 0.6% in 2011). Formal education was strongly associated with communicable disease reporting, while non-manual occupation was associated with NCD-reporting, particularly in the rainy season. |
| 37 | PUBMED/MEDLINE | GP Kynast-Wolf, M.; Sié, A.; Kouyaté, B.; Becher, H. | Seasonal patterns of cardiovascular disease mortality of adults in Burkina Faso, West Africa | 2010 | Burkina Faso | Quantitative | To evaluate seasonal patterns of cardiovascular death in adults, which are possibly influenced by hot and dry climate, in a rural setting of Burkina Faso. | Among 11,174 adults aged 40 and above, 1238 deaths occurred from 1999 to 2003. All-cause mortality rates were 1269 per 100,000 (95% CI 1156–1382) for adults aged 40–64 and 7074 (95% CI 6569–7579) for those 65 and older. Cardiovascular mortality ranked fourth among causes of death for adults 40 and older, with rates of 109.9 (95% CI 76.6–143.1) for ages 40–64 and 544.9 (95% CI 404.6–685.1) for ages 65 and older. Mortality peaked in March overall and in April specifically for cardiovascular deaths, coinciding with the hot dry season (March–May). Mean monthly temperature showed a significant association with mortality in older age groups. |
| 38 | SCOPUS | TCW Adebayo-Ojo, J.; Arowosegbe, OO; Probst-Hensch, N.; Schindler, C.; Kunzli, N. | Short-term effects of PM10, NO2, SO2 and O3 on cardio-respiratory mortality in Cape Town, South Africa, 2006–2015 | 2022 | South Africa | Time-series analysis | To estimate the associations of daily concentrations of PM10, NO2, SO2, O3 with daily mortality due to respiratory and cardiovascular diseases, using both single and multi-pollutant models. | Increased daily average concentrations of air pollutants, including PM10, NO2, SO2, and O3, were positively associated with CVD, with risk increases of 2.4% (95% CI: 0.9–3.9%), 2.2% (95% CI: 0.4–4.1%), 1.4% (95% CI: 0–2.8%), and 2.5% (95% CI: 0.2–4.8%) per interquartile range (IQR) increase, respectively. Only NO2 showed a significant positive association with respiratory disease (RD), with a 4.5% (95% CI: 1.4–7.6%) increase per IQR. In multi-pollutant models, NO2 associations with RD remained unchanged when adjusted for PM10 and SO2 but were weakened for O3. O3 estimates for CVD were insensitive to other pollutants, indicating an increased risk. Notably, PM10 showed significant acute effects on both CVD and RD with evidence of mortality displacement. |
| 39 | PUBMED/MEDLINE | Barrak Alahmad, MD, MPH, PhD; Haitham Khraishah, MD; Dominic Royé, PhD; Ana Maria Vicedo-Cabrera, PhD; Yuming Guo, PhD; Stefania I. Papatheodorou, MD; Souzana Achilleos, ScD; Fiorella Acquaotta, PhD; Ben Armstrong, PhD; Michelle L. Bell, PhD; Shih-Chun Pan, PhD; Micheline de Sousa Zanotti Stagliorio Coelho, PhD; Valentina Colistro, PhD; Tran Ngoc Dang, PhD; Do Van Dung, PhD; Francesca K. De'Donato, PhD; Alireza Entezari, PhD; Yue-Liang Leon Guo, PhD; Masahiro Hashizume, PhD; Yasushi Honda, PhD; Ene Indermitte, PhD; Carmen Íñiguez, PhD; Jouni JK Jaakkola, PhD; Ho Kim, PhD; Eric Lavigne, PhD; Whanhee Lee, PhD; Shanshan Li, PhD; Joana Madureira, PhD; Fatemeh Mayvaneh, PhD; Hans Orru, PhD; Ala Overcenco, PhD; Martina S. Ragettli, PhD; Niilo RI Ryti, PhD; Paulo Hilario Nascimento Saldiva, PhD; Noah Scovronick, PhD; Xerxes Seposo, PhD; Francesco Sera, PhD; Susana Pereira Silva, MSc; Massimo Stafoggia, PhD; Aurelio Tobias, PhD; Eric Garshick MD; Aaron S. Bernstein, MD; Antonella Zanobetti, PhD; Joel Schwartz, PhD; Antonio Gasparrini, PhD; Petros Koutrakis, PhD | Associations between extreme temperatures and cardiovascular cause-specific mortality: results from 27 countries | 2023 | Other countries | Quantitative | To investigate associations between extreme temperatures and all CVD causes, ischemic heart disease, stroke, heart failure, and arrhythmia using a standardized analytic protocol. | The analysis covered deaths from various cardiovascular causes: any cardiovascular cause (32,154,935), ischemic heart disease (11,745,880), stroke (9,351,312), heart failure (3,673,723), and arrhythmia (670,859). Extreme temperatures, both heat (99th percentile) and cold (1st percentile), were linked to higher risks of mortality from any cardiovascular cause, ischemic heart disease, stroke, and heart failure compared to the minimum mortality temperature. On extreme hot days (above 97.5th percentile) and cold days (below 2.5th percentile), there were 2.2 (95% eCI: 2.1–2.3) and 9.1 (95% eCI: 8.9–9.2) excess deaths per 1000 cardiovascular deaths, respectively. Heart failure had the highest proportion of excess deaths on extreme hot and cold days, with 2.6 (95% eCI: 2.4–2.8) and 12.8 (95% eCI: 12.2–13.1) excess deaths per 1000 heart failure deaths, respectively. |
| 40 | SCOPUS | Zunnunov, Z.R.; | The central hemodynamic reaction of patients with ischemic heart disease to the climatic and weather conditions of an arid zone | 1991 | Other countries | Qualitative | To evaluate the central hemodynamic reaction of patients with ischemic heart disease to the climatic and meteorological conditions of an arid zone | Before the invasion of the dust storm, atmospheric conditions were hypoxic. On the day of the storm, the weather appeared spastic, resulting in arterial hypertension, elevated total peripheral resistance, and decreased cardiac output and stroke volume. Hot weather brought about a hypotensive response, with total peripheral resistance falling, cardiac output increasing at the expense of enhanced heart rate as stroke volume decreased. |
| 41 | SCOPUS | Morris, R.D.; | Airborne particulates and hospital admissions for cardiovascular disease: a quantitative review of the evidence | 2021 | Other countries | Narrative review | To characterize the relationship between exposure to airborne particles and hospital admissions for cardiovascular diseases. | The results were grouped and compared based on specific outcomes and exposure measures. After pooling studies examining the association between PM10 exposure and specific cardiovascular outcomes (following outlier exclusion), a 10-µg/m3 increase in PM10 was associated with admission rate increases of 0.8% (95% CI: 0.5, 1.2%) for congestive heart failure, 0.7% (95% CI: 0.4, 1.0%) for ischemic heart disease, and 0.2% (95% CI: -0.2, 0.6%) for cerebrovascular accidents. These effects tended to decrease substantially when considering gaseous co-pollutants. |
| 42 | SCOPUS | Chang, CC; Tsai, SS; Ho, SC; Yang, C.Y.; | Air pollution and hospital admissions for cardiovascular disease in Taipei, Taiwan | 2005 | Taiwan | Quantitative | To determine whether there is an association between air pollutants levels and hospital admissions for cardiovascular diseases (CVD) in Taipei, Taiwan. | In one-pollutant models, warm days (≥20°C) showed significant positive associations between PM10, NO2, CO, and O3 levels and cardiovascular disease (CVD) admissions. On cool days (<20°C), all pollutants except O3 and SO2 were significantly associated with CVD admissions. In two-pollutant models, CO, NO2, and O3 were significant alongside each of the other four pollutants on warm days. On cool days, PM10 remained statistically significant in all two-pollutant models. |
| 43 | SCOPUS | Chan, CC; Chuang, K.J.; Chen, W.J.; Chang, W. T.; Lee, CT; Peng, CM; | Increasing cardiopulmonary emergency visits by long-range transported Asian dust storms in Taiwan | 2008 | Taiwan | Quantitative | To explore whether Asian dust storms can affect health after 4000 km long-range transport from their origins to downwind area | There were 39 high dust events with PM10 exceeding 90 μg/m3 and 46 low dust events with PM10 below 90 μg/m3. PM10 concentrations increased significantly by 77 μg/m3 per event during high dust events compared to pre-dust periods. Asian dust storms elevate cardiopulmonary emergency visits in Taipei when PM10 concentrations exceed 90 μg/m3. Emergency visits for ischemic heart diseases, cerebrovascular diseases, and COPD during high dust events increased by 0.7 cases (35%), 0.7 cases (20%), and 0.9 cases (20%) per event, respectively, according to paired t-tests. |
| 44 | SCOPUS | Linares, C.; Díaz, J.; | Impact of high temperatures on hospital admissions: comparative analysis with previous studies about mortality | 2008 | Spain | Quantitative | To investigate the impact of high temperatures on hospital admissions | The results indicate that the temperature threshold for hospital admissions corresponds to the temperature threshold for increased mortality, aligning with the 95th percentile of maximum daily temperature during summer months. However, the pattern of hospital admissions differs significantly from that of mortality. While hospital admissions for all causes and age groups increase, the rise is notably smaller compared to the increase in mortality. |
| 45 | SCOPUS | Huang, CH; Lin, H.C.; Tsai, CD; Huang, H.K.; Lian, IB; Chang, CC; | The interaction effects of meteorological factors and air pollution on the development of acute coronary syndrome | 2017 | Taiwan | Quantitative | To investigate the effects of the interaction of meteorological factors and atmospheric pollutants on the onset of acute coronary syndrome (ACS) | High temperatures (>26°C) and low atmospheric pressure (<1009 hPa) on the previous day were linked to a higher likelihood of developing ACS. Typhoon Morakot, characterized by high temperatures and extremely low atmospheric pressure, led to a higher incidence of ACS compared to daily averages. Additionally, combinations of high PM or CO concentrations with low temperatures (<21°C) and high humidity levels with low temperatures were also associated with an increased incidence of ACS. |
| 46 | SCOPUS | Chen, F.; Fan, Z.; Qiao, Z.; Cui, Y.; Zhang, M.; Zhao, X.; Li, X.; | Does temperature modify the effect of PM10 on mortality? A systematic review and meta-analysis | 2017 | Other countries | Systematic review | To review information from 29 studies to obtain qualitative evidence of the effects of temperature modification on PM on mortality, and perform a meta-analysis. | The effect of PM10 on respiratory death was the greatest, while the effect on non-accidental death was the smallest at the same temperature level. In addition, the effects of PM10 on all three types of mortality were the biggest in the high-temperature level, and the smallest in the low-temperature level. |
| 47 | SCOPUS | Li, J.; Woodward, A.; Hou, XY; Zhu, T.; Zhang, J.; Brown, H.; Yang, J.; Qin, R.; Gao, J.; Gu, S.; Li, J.; Xu, L.; Liu, X.; Liu, Q.; | Modification of the effects of air pollutants on mortality by temperature: A systematic review and meta-analysis | 2017 | Other countries | Systematic review | To summarize the epidemiological evidence on temperature modification of the acute effects of air pollutants on non-accidental and cardiovascular mortality | The effect of O3 on cardiovascular mortality was strongest on high temperature days with pooled estimate of 1.63% (1.14%, 2.13%). No significant interactions between SO2/NO2 and temperature were detected by meta-analysis. Other pollutants were not analyzed due to the lack of suitable studies. In summary, we observed interactions between high temperature and PM10 and O3 in the effects on non-accidental and cardiovascular mortality. |
| 48 | SCOPUS | Jiang, J.; Niu, Y.; Liu, C.; Chen, R.; Cao, J.; Kan, H.; Cheng, Y.; | Short-term exposure to coarse particulate matter and outpatient visits for cardiopulmonary disease in a Chinese city | 2020 | China | Quantitative | To explore the relationship of PM2.5-10 with outpatient visits for cardiopulmonary diseases. | PM2.5-10 exposure with a lag of 05 days yielded the best estimates for both outcomes. A 10-μg/m3 increase in PM2.5-10 was associated with a 1.69% (95% CI: 0.68%–2.72%) increase in outpatient visits for respiratory causes and a 0.85% (95% CI: 0.13%–1.57%) increase for cardiovascular causes. The association remained robust after adjusting for PM2.5 and O3, with larger associations observed in warm seasons. Concentration-response curves had a steeper slope for respiratory diseases at relatively lower concentrations (<30 μg/m3), while PM2.5-10 was positively associated with cardiovascular diseases at higher concentrations (>30 μg/m3). |
| 49 | SCOPUS | Li, X.; Cai, H.; Ren, X.; He, J.; Tang, J.; Xie, P.; Wang, N.; Nie, F.; Lei, L.; Wang, C.; Li, W.; Ma, J.; | Sandstorm weather is a risk factor for mortality in ischemic heart disease patients in the Hexi Corridor, northwestern China | 2020 | China | Times series | To explore the association between sandstorm weather and ischemic heart disease (IHD)-related mortality in this area. | The results revealed a higher frequency of sandstorms in the Hexi Corridor compared to control regions (5.48% vs 1.64%, P < 0.01), accompanied by higher IHD mortality rates (56.42/100,000 vs 45.62/100,000, P < 0.01). Stratification by gender, age, and urban/rural residence also showed significant differences in IHD-related mortality (P < 0.05). There were significant associations between sandstorm weather and IHD-related mortality, with the relative risk (RR) increasing with the number of sandstorm days. Monthly and annual analyses revealed mortality rates corresponding to sandstorm frequency. These findings suggest a positive association between sandstorm weather and IHD-related mortality in the Hexi Corridor of Gansu Province. |
| 50 | SCOPUS | Lokotola, CL; Wright, C.Y.; Wichmann, J.; | Temperature as a modifier of the effects of air pollution on cardiovascular disease hospital admissions in Cape Town, South Africa | 2020 | South Africa | Quantitative | To investigate the interaction and potential modification of each other's effect in developing countries. | In total, 54,818 CVD hospital admissions were included in the study. In general, on warm and cold days the 15–64 years old group was more at risk for CVD hospitalization with increasing air pollution levels compared to all ages combined or the ≥ 65 years old group. Females appeared to be more at risk than males with increasing PM10 levels. In contrast, males were more vulnerable to the effects of NO2 and SO2 than females. The study showed the modification effect of temperature on air pollution associated with CVD hospital admissions. |
| 51 | SCOPUS | Vered, S.; Paz, S.; Negev, M.; Tanned.; Zucker, I.; Weinstein, G.; | High ambient temperature in summer and risk of stroke or transient ischemic attack: A national study in Israel | 2020 | Israel | Quantitative | To examine whether high ambient temperature and diurnal temperature range during the summer are associated with risk of stroke/transient ischemic attack (TIA) | The study included 15,123 individuals with stroke/TIA during summer (mean age 73 ± 12 years; 54% males). High ambient temperature increased stroke/TIA risk starting from the day before the event, intensifying over a six-day lag (OR = 1.10, 95% CI: 1.09–1.12). A larger diurnal temperature range prior to stroke/TIA occurrence was associated with decreased risk (OR = 0.96, 95% CI: 0.95–0.97 for a six-day lag). |
| 52 | SCOPUS | Xu, ZW; Tong, S.L.; Hu, WB; Cheng, J.; Huang, CR; Zheng, H.; | Impact of heatwave definition on health effect assessment: evidence from Thailand | 2020 | Thailand | Quantitative | To understand the impact of heatwave definition on the assessment of the health effects of heatwaves, and to provide evidence for the development of heatwave early warning systems. | During heatwave periods, the risks of total and cause-specific deaths significantly increased compared to non-heatwave periods. Mild heatwaves (>90th percentile of mean temperature and ≥2 days) saw a 12.8% increase in total deaths (95% CI: 10.6%-15.1%). However, the relative risk (RR) of deaths during heatwaves did not consistently increase with higher intensity or duration. For heatwaves lasting ≥2 days, pneumonia-related deaths (RR: 1.42, 95% CI: 1.27-1.59) and diabetes-related deaths (RR: 1.34, 95% CI: 1.17-1.52) were most vulnerable when defined by the 97th percentile of mean temperature. Meanwhile, total deaths (RR: 1.16, 95% CI: 1.13-1.20) and deaths due to ischemic heart disease (RR: 1.35, 95% CI: 1.24-1.48) were most vulnerable when using the 95th percentile. When the heatwave cut-off was the 98th percentile, diabetes-related deaths increased with longer durations, while total deaths and pneumonia-related deaths decreased. |
| 53 | PUBMED/MEDLINE | Manochehrneya, S.; Mohammadi, M.; Esmaeili, R.; Vahdani, A.; | A time series approach to estimate the association between health effects, climate factors and air pollution, Mashhad, Iran | 2020 | Iran | Times series | To evaluate the correlation between climatic parameters and air pollution with cardiovascular disease and its associated death during 2014 in Mashhad by time series model. | The monthly survey revealed significant correlations (p < 0.05) between humidity (positive), temperature (positive), wind speed (negative), and PM2.5 (negative) with average values ​​of 16.2471, 48.1628, 122.38, and 7.3905, respectively, on the number of people experiencing cardiovascular disease. Meanwhile, the mortality rate due to cardiovascular disease showed significant correlations (p < 0.05) with pressure (positive), temperature (negative), and rainfall (negative), with average values ​​of 6.5904, 1.5728, and 1.1704, respectively. Notably, there was a significant difference in the numbers of cardiovascular disease patients across different seasons, with the highest recorded number of 3778 in autumn. |
| 54 | PUBMED/MEDLINE | Kalankesh, L.R.; Rodríguez-Couto, S.; Alami, A.; Khosravan, S.; Meshki, M.; Ahmadov, E.; Mohammadpour, A.; Bahri, N.; | Socio-environmental determinants and human health exposures in arid and semi-arid zones of Iran-narrative review | 2022 | Iran | Narrative review | To investigate socio-environmental determinants and human health exposures in arid and semi-arid areas of Iran | The population in Iran's arid and semi-arid zones faces respiratory, cardiovascular, cancer, and infection diseases due to environmental issues like air and water pollution. Specifically, skin, stomach, bladder, prostate, and colorectal cancers, along with respiratory and cardiovascular diseases, are more prevalent in areas like Kerman and Yazd compared to other provinces. These health impacts affect multiple levels of health security in these zones. |
| 55 | PUBMED/MEDLINE | Yan-Ru, Wang; Ji-Yuan, Dong; Ren-Qing, Yang; Ning, LIU; | Air temperature affects the hospital admission for cardiovascular diseases among rural residents in Dingxi City | 2022 | China | Quantitative | To explore the effect of air temperature on the hospitalization of rural residents with cardiovascular diseases and its lag effect in Dingxi city. | There was a non-linear relationship between air temperature and cardiovascular hospitalizations in Dingxi city, resembling a bell shape. Low temperatures (-7 ℃) and moderately low temperatures (0 ℃) had significant cumulative lag effects, peaking at 7 and 14 days respectively. High temperatures (17 ℃ and 21 ℃) also had significant cumulative lag effects on certain cardiovascular diseases, peaking on the same day. Notably, the impact of temperature on hospitalizations differed between genders, with higher sensitivity observed in females. Additionally, individuals under 65 years old were more sensitive to temperature fluctuations than those aged 65 and above. |
| 56 | SCOPUS | Rodríguez, D.; Cobo-Cuenca, AI; Quiles, R.; | Effects of air pollution on daily hospital admissions for cardiovascular diseases in Castilla-La Mancha, Spain: a region with moderate air quality | 2022 | Spain | Times series | To assess the relationship between the risk of hospital admission for cardiovascular disease (CVD) and exposure to PM2.5, PM10, and NO2 in CLM during 2006–2015. | Daily air pollution concentrations, temperature, and relative humidity were monitored in CLM. A time-series analysis using generalized linear models examined the effects of air pollution on hospital admissions, controlling for long-term trends and potential confounders. Effect modifications by sex and age (15–64; ≥65 years) were assessed. Lagging exposure was utilized to explore potential latency periods in cumulative exposure-pollution analyses. Relative risks (RR) of cardiovascular disease (CVD) admissions were calculated for exposure at the same day (lag 0) and up to 7 days after exposure (lag 7). The group of older adults (≥65 years) showed the highest susceptibility to air pollution, while the effect varied significantly by sex depending on the age group. |
| 57 | PUBMED/MEDLINE | Rahman, M.M.; McConnell, R.; Palinkas, L.; Johnston, J.; Hurlburt, M.; Schlaerth, H.; Ko, J.; Silva, S.; Lurmann, F.W.; Yin, Hao; Ban-Weiss, G.; Garcia, E.; | The effects of coexposure to extremes of heat and particulate air pollution on mortality in California: implications for climate change | 2022 | USA | Quantitative | To estimate the association of acute coexposure to extreme heat and ambient fine particulate matter (PM2.5) with all-cause, cardiovascular, and respiratory mortality in California from 2014 to 2019. | The risk of all-cause mortality increased by 6.1% (95% CI: 4.1-8.1) on extreme maximum temperature-only days and by 5.0% (95% CI: 3.0-8.0) on extreme PM2.5-only days, compared to non-extreme days. There was a 21.0% (95% CI: 6.6-37.3) increased risk on days with exposure to both extreme maximum temperature and PM2.5. The increased risk of cardiovascular and respiratory mortality on extreme co-exposure days was 29.9% (95% CI: 3.3-63.3) and 38.0% (95% CI: -12.5 to 117.7), respectively, exceeding the sum of individual effects of extreme temperature and PM2.5 alone. A similar pattern was observed for co-exposure to extreme PM2.5 and minimum temperature. Effect estimates were larger for individuals over 75 years old. |
| 58 | PUBMED/MEDLINE | HK Armenian, AK Melkonian, AP Hovanesian | Long term mortality and morbidity related to degree of damage following the 1988 earthquake in Armenia | 1998 | Armenia/USA | Longitudinal | To assess the relationship of increased mortality and morbidity to personal loss and damage following the 1988 earthquake in Armenia | The study suggests that the intensity of exposure to disaster-related damage and losses correlates with longer-term increased rates of heart disease and chronic disease morbidity following an earthquake. It emphasizes the importance of closely monitoring individuals who sustain such losses for heightened long-term morbidity. |
| 59 | SCIELO | Sanhueza H, Pedro; Vargas R, Claudio; Mellado G, Paula; | Impact of air pollution by fine particulate matter (PM10) on daily mortality in Temuco, Chile | 2006 | Chile | Time series analysis | To investigate the short-term effects of PM 10 on daily mortality in Temuco, in Southern Chile, due to respiratory and cardiovascular causes. | There was a significant and positive association between PM 10 concentration and daily mortality caused by respiratory disease (p-value=0.046, relative risk (RR) 1.236, 95% confidence interval (CI) 1.004-1.522) and cardiovascular diseases in aged people 65 years and more (p-value=0.042; RR 1.176 95% CI 1.006-1.374). |
| 60 | INSTITUTE OF ELECTRICAL AND ELECTRONICS ENGINEERS | Elkadhi, H.; Ben Hamida, R.; | The short-term effects of air pollution on health in Sfax (Tunisia): an ARDL cointegration procedure | 2014 | Tunisia | Qualitative | To explore the short-term causal link between air pollution and the frequency of cardiovascular and respiratory diseases in urban area of ​​Sfax | The results show that there is a significant link between pollutant emissions especially sulfur dioxide (SO2) and the ozone (O3) and hospital admissions for cardiovascular and respiratory diseases. |
| 61 | PUBMED/MEDLINE | Ebrahimi, SJA; Ebrahimzadeh, L.; Eslami, A.; Bidarpoor, F.; | Effects of dust storm events on emergency admissions for cardiovascular and respiratory diseases in Sanandaj, Iran | 2014 | Iran | Time series analysis | To evaluate possible effects of dust storms on incidence of cardiovascular and respiratory diseases among residents of Sanandaj. | The average PM10levels during dust episodes (187 μg/m3) were significantly higher than the other days (48.7 μg/m3). In addition, correlation coefficient between PM10level and number of cardiovascular and emergency services during dust events were equal to 0.48 (P <0.05) and 0.19 (P >0.05) respectively. |
| 62 | SCOPUS | Ryan B, Franklin RC, Burkle FM, Aitken P, Smith E, Watt K, Leggat P | Identifying and describing the impact of cyclone, storm and flood related disasters on treatment management, care and exacerbations of non-communicable diseases and the implications for public health | 2015 | Australia | Systematic review | To perform a systematic review that identifies and describes the impact of cyclone, flood and storm related disasters on those susceptible to, or experiencing, NCDs. | The review identified 48 relevant articles, all based on data from developed countries. Disasters interrupted treatment management and overall care for individuals with NCDs, leading to heightened risk of exacerbation or death. Factors such as damaged transport routes, reduced health services, power loss, and evacuations contributed to these interruptions. The health impact varied by NCD type: individuals with chronic respiratory diseases faced an increased risk of acute exacerbation, while those with cancer, cardiovascular diseases, and diabetes were at risk of worsening illness that could lead to death. |
| 63 | EBSCO | Resnick, Adam; Woods, Brian; Krapfl, Heidi; Toth, Barbara; | Health outcomes associated with smoke exposure in Albuquerque, New Mexico, during the 2011 Wallow Fire | 2015 | Mexico | Poisson regression analysis | To examine the association between PM2.5 levels and emergency department (ED) visits for selected health outcomes in Albuquerque, New Mexico, during the Wallow fire of 2011. | Analysis of PM2.5 exposure data and ED visits in Albuquerque before and during the Wallow fire revealed an increased risk of ED visits for respiratory and cardiovascular conditions during heavy smoke conditions, with variations by age and sex. The population aged 65 and above was particularly vulnerable, showing significantly increased risks for asthma (RR = 1.73, 95% CI = 1.03-2.93) and diseases of the veins, lymphatic, and circulatory system (RR = 1.56, 95% CI = 1.00-2.43). In the 20 to 64 age group, there were statistically significant increases in ED visits for diseases of pulmonary circulation (RR = 2.64, 95% CI = 1.42-4.9) and cerebrovascular disease (RR = 1.69, 95% CI = 1.03-2.77). The findings underscore the association between high PM2.5 exposure from the Wallow fire and increased ED visits for respiratory and cardiovascular conditions in Albuquerque. |
| 64 | SCOPUS | Soleimani, Z.; Darvishi Boloorani, A.; Khalifeh, R.; Griffin, D.W.; Mesdaghinia, A.; | Short-term effects of ambient air pollution and cardiovascular events in Shiraz, Iran, 2009 to 2015 | 2019 | Iran | Qualitative | To investigate the association between short-term exposure to ambient air pollutants and CVD (cardiovascular disease) events in a long-term observational period. | The highest association of each pollutant with hospital admission was observed as PM 10 at lag 4 (RR = 1.08; 95% CI 1.02, 1.14 and p < 0.05), NO 2 at lag 0 (RR = 1.22; 95% CI 1.00, 1.48 ), and CO at lag 0 (RR = 1.52 95% CI = (1.16, 1.99)). However, on dusty days, there were significantly higher numbers of referrals of cardiovascular patients (mean = 7.54 ± 4.44 and p = 0.002) than on non-dusty days. According to these data, dust storms and some types of pollutants in the air are responsible for more admissions to hospitals for cardiovascular problems. |
| 65 | PUBMED/MEDLINE | Troy Quast, Ross Andel, and Archana R. Sadhu | Long-term effects of disasters on health care utilization: Hurricane Katrina and older individuals with diabetes | 2019 | USA | Retrospective cohort analysis | To etimate the long-run mortality effects of Hurricanes Katrina and Rita on seniors with diabetes. | The affected subjects had a nearly 40% higher all-cause mortality risk in the 1st month after the storms, but the difference fell to <6% by the end of the full observation period. The mortality risks of heart disease and nephritis also exhibited the largest differences immediately following the storms. Among the affected subjects, the all-cause mortality risk was higher for those who moved to a different county, with an especially large difference among those who moved to an affected county. |
| 66 | SCOPUS | Leili M, Nadali A, Karami M, Bahrami A, Afkhami A | Short-term effect of multi-pollutant air quality indexes and PM2.5 on cardiovascular hospitalization in Hamadan, Iran: a time-series analysis | 2021 | Iran | Time-series analysis | To investigate the number of hospitalizations due to heart failure (HF) and myocardial infarction (MI) following the air pollutant exposure using a time-series regression analysis with a distributed lag model in Hamadan, Iran (2015-2019). | A total of 2091 cases of CVD were registered. Results showed that hospitalization in the warm season was higher than that of the cold season. |
| 67 | PUBMED/MEDLINE | Sepandi, M.; Akbari, H.; Naseri, MH; Alimohamadi, Y.; | Emergency hospital admissions for cardiovascular diseases attributed to air pollution in Tehran during 2016-2019 | 2021 | Iran | Time-series | To assess the burden of five major air pollutants, including CO, O3, NO2, SO2 and PM2.5, in emergency department visits (EDVs) during January 2016 to December 2019 due to all cardiovascular diseases in Tehran | About 3800 emergency department visits were significantly attributed to CO, of which over 3000 were significantly attributed to high values ​​of the pollutant. |
| 68 | PUBMED/MEDLINE | Sokoty, L.; Rimaz, S.; Hassanlouei, B.; Kermani, M.; Janani, L.; | Short-term effects of air pollutants on hospitalization rate in patients with cardiovascular disease: a case-crossover study | 2021 | Iran | Case-crossover study | To determine the relationship between the amount of atmospheric pollutants and the incidence of cardiovascular diseases that lead to hospitalization. | The CO for each increase of 10 μg/m3 had a significant relationship with the incidence of cardiovascular hospitalization. |
| 69 | PUBMED/MEDLINE | Ye, T.; Guo, Y.; Chen, G.; Yue, X.; Xu, R.; Coêlho, Mszs; Saldiva, PHN; Zhao, Q.; Li, S.; | Risk and burden of hospital admissions associated with wildfire-related PM(2·5) in Brazil, 2000-15: a nationwide time-series study | 2021 | Brazil | Time-series | To estimate the association between daily exposure to forest fire-related PM(2·5) and cause-specific hospital admission and attributable health burden in the Brazilian population using a national dataset from 2000 to 2015. | Short-term exposure to wildfire-related PM(2·5) has been associated with increased risks of all-cause hospital admissions, respiratory and cardiovascular, particularly among children (0-9 years) and older people (≥80 years). |
| 70 | PUBMED/MEDLINE | Sadeghimoghaddam, A.; Khankeh, H.; Norozi, M.; Fatah, S.; Farrokhi, M.; | Effects of dust events and meteorological elements on stroke morbidity in northern Khuzestan, Iran | 2021 | Iran | Retrospective cohort study | To assess the effects of dust events and meteorological elements on health stroke morbidity in Iran (a health promotion approach). | Increasing severity of the dust event increases the risk of stroke in men. Increasing average wind speed also increases the risk of stroke in men. Increased rainfall and average relative humidity increase the risk of stroke in people under 60 years of age. Increasing average daily temperature reduces stroke risk in men. |
| 71 | PUBMED/MEDLINE | Aghababaeian, H.; Sharafkhani, R.; Kiarsi, M.; Mehranfar, S.; Moosavi, A.; Araghi Ahvazi, L.; Aboubakri, O.; | Diurnal temperature range and hospital admission due to cardiovascular and respiratory diseases in Dezful, a city with hot climate and high DTR fluctuation in Iran: an ecological time-series study | 2023 | Iran | Time-series | To investigate the effects of daytime temperature range (DTR) and hospital admission on cardiovascular and respiratory diseases in Dezful, Iran | Extremely low daytime temperature range can increase the risk of daily cardiovascular admissions and extremely high levels. Diurnal temperature ranges may have a protective effect on daily respiratory and cardiovascular admissions in some regions with high fluctuations in the diurnal temperature range. |
| 72 | PUBMED/MEDLINE | Martinez-Lozano, M.; Noboa, C.; Alvarado-Gonzalez, G.; Joshipura, K.J.; | Hurricanes Irma and Maria and diabetes incidence in Puerto Rico | 2023 | Puerto Rico | Longitudinal Study | To evaluate the impact of Hurricanes Irma/Maria on diabetes incidence in Puerto Rico. Mortality increased substantially after the hurricanes, but morbidity was not assessed. | The incidence of diabetes was significantly higher during hurricanes than before. |
| 73 | PUBMED/MEDLINE | Nhung, NTT; Hoang, L.T.; Tuyet Hanh, T.T.; Toan, L.Q.; Thanh, N.D.; Truong, NX; Son, NA; Nhat, HV; Quyen, NH; Nhu, HV | Effects of heatwaves on hospital admissions for cardiovascular and respiratory diseases, in Southern Vietnam, 2010–2018: time series analysis | 2023 | Vietnam | Time-series analysis | To investigate the associations between heatwaves and daily hospital admissions for cardiovascular and respiratory diseases in two provinces in Vietnam known to be vulnerable to droughts during 2010–2018 | Heatwaves were negatively associated with cardiovascular disease in Ca Mau, which was determined among the older adults (age above 60 years), ER = −7.28%, 95% CI: −13.97–−0.08 %. |
| 74 | SCOPUS | Rawal, H.; Nakhle, A.; Peters, M.; Srivastava, A.; Srivastava, S.; Irimpen, A.; | Incidence of acute myocardial infarction and hurricane Katrina: fourteen years after the storm | 2023 | Other countries | Retrospective observational study cohort | To study whether the effects of Hurricane Katrina on the incidence of acute myocardial infarction (AMI) were perpetuated or mitigated after the first decade. | The pre-Katrina cohort had an AMI incidence of 0.7%, while the post-Katrina cohort had an AMI incidence of 3.0% (p < 0.001). The post-Katrina group also had significantly higher comorbidities, including diabetes, hypertension, polysubstance abuse, and coronary artery disease. |

**Table S14: Overview of cardiovascular diseases/diabetes, associated risk factors, gender, age, and climate variables examined in the studies**

| **ID** | **Cardiovascular diseases/Diabetes** | **Risk factors** | **Gender** | **Age** | **Climate variables** |
| --- | --- | --- | --- | --- | --- |
| 1 | Cardiovascular Diseases | Older adults, people with respiratory, kidney or heart diseases, diabetes, children, residents of urban areas and physical exercise | Men and women | 0-1 year and 65 years | Temperature |
| 2 | Cardiovascular Diseases | Exposure to dry areas | Not specified | Not specified | Humidity |
| 3 | Stroke | Dust exposure | Not specified | Not specified | Temperature, humidity and precipitation |
| 4 | Cardiovascular Diseases | Exposure to dust | Not specified | >, = 65 years | Wind |
| 5 | Cardiovascular Diseases | Exposure to dust | Men and women | >, = 65 years | Temperature and wind |
| 6 | heart disease | Exposure to natural disasters | Not specified | 0–9 years, 10–19 years, 20–59 years, ≥ 60 years | Not specified |
| 7 | Myocardial infarction | Such as tobacco use, the harmful use of alcohol, socioeconomic status, natural disasters, and exposure to extreme temperatures, particulate matter (PM). Hypertension, increased BMI, high blood glucose, high-sodium diets and a lower socioeconomic level, inactivity | Men and women | Not specified | Temperature |
| 8 | Stroke | Natural disasters (earthquakes, extreme weather conditions), foods, viral pandemics, such as hypertension, high glucose, high total cholesterol and smoking, difficulty accessing medical services | Men and women | Not specified | Not specified |
| 9 | Myocardial infarction and diabetes | Exposure to natural disasters | Not specified | Not specified | Not specified |
| 10 | Myocardial infarction, stroke and congestive heart failure | Exposure to air pollutants | Not specified | Not specified | Temperature |
| 11 | Cardiovascular Diseases | Exposure to heat and extreme weather events | Not specified | <5-65 years | Temperature |
| 12 | coronary syndrome | Long-term exposure to dust storms, advanced age, children, pregnant individuals, older adults, those reporting lower levels of income, and those with preexisting cardiovascular and respiratory diseases | Women | > 65 years | Wind |
| 13 | Cardiovascular Diseases | Exposure to air pollutants | Not specified | Not specified | Temperature |
| 14 | Coronary heart disease | Exposure to episodes, concomitant poor air quality, dry, urban areas | Not specified | > 60 years | Temperature and precipitation |
| 15 | Cardiovascular Diseases | Exposure to drought and exposure in periods of drought aggravation | Not specified | 65 years or older | Not specified |
| 16 | Myocardial infarction | Exposure to particulate matter (PM) | Not specified | > 30 years and > 65 years | Not specified |
| 17 | Cardiovascular Diseases | Exposure to dust storms | Not specified | 15-64 years | Temperature and humidity |
| 18 | Stroke | Exposure to air pollutants | Not specified | Not specified | Humidity |
| 19 | Cardiovascular Disease | Exposure to particulate matter (PM) | Not specified | > 30 years and > 65 years to 99, = OR > 75, 0-17 years | Not specified |
| 20 | Hypertension | Exposure to natural disasters | Men and women | 13-17 years | Not specified |
| 21 | Cardiovascular Diseases | Exposure to landscape fires and atmospheric pollutants | Not specified | Not specified | El niño |
| 22 | Cardiovascular diseases, hypertension and diabetes | Stress, lack of health insurance, interruption of treatment | Both, but mostly on women | 65-74, 75-84 and 85 years | Not specified |
| 23 | Cardiovascular Diseases | Extreme weather events | Not specified | Not specified | Temperature |
| 24 | Cardiovascular Diseases | Exposure to particulate matter (PM)* | Not specified | Not specified | Wind |
| 25 | Cardiovascular Diseases | Extreme weather events and general weather conditions | Not specified | Not specified | Temperature and humidity |
| 26 | Heart Failure and Diabetes | Chronic diseases | Not specified | 60 years or older | Not specified |
| 27 | Diabetes mellitus | Stress, lack of medication | Not specified | Not specified | Not specified |
| 28 | Acute coronary syndromes, Myocardial infarction | Exposure to high temperatures and hot flashes | Not specified | Not specified | Temperature and humidity |
| 29 | Myocardial infarction and arrhythmias | Exposure to fumes from forest fires, | Not specified | > 65 years | Temperature |
| 30 | Myocardial infarction | Exposure to dust | Mostly men | 45-64 and, 74 years | Temperature and wind |
| 31 | Cardiovascular diseases and Diabetes mellitus | Relocation after hurricanes and storms | Women | Not specified | Not specified |
| 32 | Diabetes mellitus | Hypertension and cholesterol, difficult access to drugs | Not specified | equal or elder than 15 years of age | Not specified |
| 33 | Cardiovascular Diseases | Exposure to extreme temperatures and disasters, Low income countries. | Not specified | > 65 years | Temperature and precipitation |
| 34 | Myocardial infarction, Coronary artery disease | Smoking and hyperlipidemia, lack of health insurance, unemployment, substance abuse and psychiatric illnesses | Men | 64 +/- 14 | Not specified |
| 35 | Diabetes mellitus and hypertension | Exposure to natural disasters, chronic illnesses | Not specified | Not specified | Not specified |
| 36 | Hypertension and Chronic Heart Disease | Exposure during the rainy season - seasonality | Women | > 65 years | Not specified |
| 37 | Cardiovascular Diseases | Exposure during the dry season - seasonality | Men and women | 40 years and > 65 years | Temperature |
| 38 | Cardiovascular Diseases | Exposure to air pollutants - PM10 | Women | >, = 65 years | Temperature and humidity |
| 39 | Ischemic heart disease, stroke, heart failure, and arrhythmia | Exposure to temperature extremes, iow income areas | Not specified | Not specified | Temperature |
| 40 | Coronary heart diseases | Exposure to dust storms | Not specified | 40-75 years | Temperature |
| 41 | Not specified | Exposure to atmospheric pollution | Not specified | Not specified | Wind |
| 42 | Not specified | Exposure to atmospheric pollution and temperature extremes | Not specified | Not specified | Temperature |
| 43 | Ischemic heart disease and stroke | Exposure to dust storms | Not specified | Not specified | Wind |
| 44 | Not specified | Exposure to high temperatures and heat waves | Not specified | All age groups | Temperature |
| 45 | Acute coronary syndrome | Exposure to high temperatures | Not specified | Not specified | Temperature and humidity |
| 46 | Not specified | Exposure to high temperatures | Not specified | Not specified | Temperature |
| 47 | Not specified | Exposure to temperature extremes | Not specified | Not specified | Temperature |
| 48 | Not specified | Exposure to high temperatures | Not specified | Not specified | Temperature |
| 49 | Coronary heart diseases | Exposure to dust storms | Not specified | Not specified | Wind |
| 50 | Not specified | Exposure to temperature extremes | Men | > 65 years | Temperature and humidity |
| 51 | Stroke | Exposure to high temperatures | Men | 12-73 years | Temperature |
| 52 | Ischemic heart disease and diabetes | Exposure to hot flashes | Not specified | Not specified | Temperature and humidity |
| 53 | Not specified | Exposure to atmospheric pollution | Not specified | Not specified | Temperature, humidity and wind |
| 54 | Not specified | Exposure to atmospheric pollution | Not specified | Not specified | Not specified |
| 55 | Ischemic heart disease and stroke | Exposure to temperature extremes | Men | All age groups | Temperature |
| 56 | Not specified | Exposure to atmospheric pollution | Men and women | >=65 years | Temperature and humidity |
| 57 | Not specified | Exposure to temperature extremes and atmospheric pollution | Not specified | 75 years | Temperature |
| 58 | Diabetes mellitus, Hypertension, Arthritis | Alcoholism, Smoking, Physical Exercise, Age, Body Mass Index, Education, Sex. | Men | Over 70 years/21-30 years | Not specified |
| 59 | Cardiovascular Diseases | Age structure, differences in age-specific risks and differences in genetics. | Not specified | Over 65 years | Temperature and humidity |
| 60 | Cardiovascular Diseases | Personal habits (alcohol, tobacco, diet, work place heavily polluted), season (cold or hot) and age of the affected population. | Not specified | Not specified | Wind speed |
| 61 | cerebrovascular accident | Previous CVD and pulmonary disease | Not specified | Not specified | Not specified |
| 62 | High blood pressure, Diabetes mellitus, high cholesterol, heart problem | Baseline neighborhood poverty, hurricane exposure, and residence in metropolitan area | Women | Not specified | Not specified |
| 63 | Diseases of the veins, lymphatic and circulatory system, cerebrovascular disease | Exposure during wildfires, advanced age, poverty, high temperature | Not specified | Over 65 years | Temperature |
| 64 | Coronary artery disease, Ischemic heart disease, Myocardial infarction, Pneumo thrombo embolism | Social and environmental variability and lifestyles; Stress; Exposure to pollutants | Men | Over 65 years | Temperature, humidity and wind |
| 65 | Acute myocardial infarction, Congestive heart failure, Ischemic heart disease, Hypertension, Stroke/transient ischemic attack, Diabetes | Age, sex, ethnicity, disrupted access to health care providers, difficulty in obtaining proper nutrition, damaged or lost medications, and challenges in monitoring glucose levels, | Females | Not specified | Not specified |
| 66 | Myocardial infarction | Particulate matter | Not specified | Not specified | Temperature, humidity and wind |
| 67 | Cardiovascular Diseases | Particulate matter | Men and women | < 65 or > 65 | Temperature, humidity and wind |
| 68 | Ischemic heart disease, hypertension, and cerebrovascular disease | Particulate matter | Men and women | Not specified | Temperature, humidity and wind |
| 69 | Cardiovascular Diseases | Particulate matter, fires | Men and women | 5 and 9 years old people aged 80 and over | Not specified |
| 70 | Stroke | Dust event | Men and women | < 60 or > 60 years | Wind, humidity and temperature |
| 71 | Cardiovascular Diseases | Extreme temerature | Men and women | Not specified | Temperature |
| 72 | Diabetes mellitus | Disasters | Men and women | ≥65 years old | Not specified |
| 73 | Cardiovascular Diseases | Heatwaves | Men and women | 6-60 and > 60 years | Humidity |
| 74 | Acute myocardial infarction | Natural disasters, diabetes, hypertension, polysubstance abuse, hyperlipidemia, smoking, psychiatric illness | Men and women | Not specified | Not specified |
