# Supplementary material for: The Influence of Climate, Atmospheric Pollution, and Natural Disasters on Cardiovascular Diseases and Diabetes Mellitus in Drylands: A Scoping Review
Source: Public Health Rev. 2024 Aug 8;45:1607300. doi: 10.3389/phrs.2024.1607300 (PMC11338784; doi:10.3389/phrs.2024.1607300)
Supplement: Supplementary file 2 [file DataSheet1.docx]

**Supplementary appendix 1**

**Table S1: MEDLINE/Pubmed search strategies applied and adapted to other databases**

| **Search** | **Query** | **Results** |
| --- | --- | --- |
| #1 | Search: "Heart Failure"[mh] OR "Heart Failure"[tiab] OR "Cardiac Failure"[tiab] OR "Heart Decompensation"[tiab] OR "Myocardial Failure"[tiab] OR "cardiac decompensation"[tiab] OR "cardiac incompetence"[tiab] OR "cardiac insufficiency"[tiab] OR "cardial decompensation"[tiab] OR "cardial insufficiency"[tiab] OR "heart insufficiency"[tiab] OR "heart decompensation"[tiab] OR "myocardial insufficiency"[tiab] OR "Cardiovascular Diseases"[mh] OR Cardiovascular Disease*[tiab] OR Cardiovascular*[tiab] OR "Heart Diseases"[mh] OR Cardiac Disease*[tiab] OR Cardiac Disorder*[tiab] OR Heart Disease*[tiab] OR Heart Disorder*[tiab] OR angiocardiopathy[tiab] OR "cardiac anomaly"[tiab] OR "cardiac disturbance"[tiab] OR Cardiopathy[mh] OR Cardiopathy[tiab] OR "heart deficiency"[tiab] OR "heart deformity"[tiab] OR "heart disorder"[tiab] OR "heart dysfunction"[tiab] OR Hypertension[mh] OR Hypertension[tiab] OR High Blood Pressure*[tiab] OR "Myocardial Infarction"[mh] OR "Cardiovascular Stroke"[tiab] OR Cardiovascular Strok*[tiab] OR Heart Attack*[tiab] OR Myocardial Infarct*[tiab] OR "Diabetes Mellitus"[mh] OR Diabetes Mellitus[tiab] OR Stroke[mh] OR Stroke*[tiab] OR Acute Cerebrovascular Accident*[tiab] OR Acute Stroke*[tiab] OR Apoplexy[tiab] OR Brain Vascular Accident*[tiab] OR Cerebral Stroke*[tiab] OR Cerebrovascular Accident*[tiab] OR "Cerebrovascular Apoplexy"[tiab] OR Cerebrovascular Stroke*[tiab] OR Coronary Disease*[tiab] OR Coronary Heart Disease*[tiab] OR "Coronary Artery Disease"[mh] OR Coronary Arterioscleros*[tiab] OR Coronary Artery Disease*[tiab] OR Coronary Atheroscleros*[tiab] OR Left Main Disease*[tiab] OR "acute cerebrovascular lesion"[tiab] OR "acute focal cerebral vasculopathy"[tiab] OR "brain attack"[tiab] OR "brain blood"[tiab] OR "flow disturbance"[tiab] OR brain insult*[tiab] OR "cerebral insult"[tiab] OR "cerebrovascular failure"[tiab] OR "cerebrovascular injury"[tiab] OR "cerebrovascular insufficiency"[tiab] OR "cerebrovascular insult"[tiab] OR "insultus cerebralis"[tiab] OR diabetic[tiab] Sort by: Most Recent | [3,788.102](https://pubmed.ncbi.nlm.nih.gov/?term=longqueryf9296b45e58bb3b20d3a&sort=date&size=200&ac=no) |
| #2 | Search: "Droughts"[mh] OR Drought*[tiab] OR arid season[tiab] OR "dry season"[tiab] OR "Natural Disasters"[mh] OR Natural Disaster*[tiab] OR "Arid Zone"[tiab] OR "Arid Soil"[tiab] OR "Semi-Arid Zone"[tiab] OR sub-humid[tiab] OR under humid[tiab] OR "atmosphere pollution"[tiab] OR "atmospheric pollution"[tiab] OR "polluted atmosphere"[tiab] Sort by: Most Recent | [64,180](https://pubmed.ncbi.nlm.nih.gov/?term=%22Droughts%22%5Bmh%5D+OR+Drought%2A%5Btiab%5D+OR+arid+season%5Btiab%5D+OR+%22dry+season%22%5Btiab%5D+OR+%22Natural+Disasters%22%5Bmh%5D+OR+Natural+Disaster%2A%5Btiab%5D+OR+%22Arid+Zone%22%5Btiab%5D+OR+%22Arid+Soil%22%5Btiab%5D+OR+%22Semi-Arid+Zone%22%5Btiab%5D+OR+sub-humid%5Btiab%5D+OR+under+humid%5Btiab%5D+OR+%22atmosphere+pollution%22%5Btiab%5D+OR+%22atmospheric+pollution%22%5Btiab%5D+OR+%22polluted+atmosphere%22%5Btiab%5D&sort=date&size=200&ac=no) |
| #3 | Search: #1 AND #2 Sort by: Most Recent | [989](https://pubmed.ncbi.nlm.nih.gov/?term=%232+AND+%233&sort=date&size=200&ac=no) |

**Table S2: Studies classified by country**

| **Country** | **Number of studies** | **Citations** |
| --- | --- | --- |
| **United States** | 12 | Achakulwisut et al., 2018; Achakulwisut et al., 2019; Bell et al., 2022; Berman et al., 2017; Cefalu et al., 2006; Croocks et al., 2016; Franchini, 2015; Far Away; Green, 2008; Peters et al., 2014; Quast et al., 2019; Quart et al., 2019; Rahman et al., 2022 |
| **Iran** | 12 | Aghababaeian et al., 2021; Dahmardeh, 2016; Gohardehi; Moslehi, 2020; Ebrahimi et al., 2014; Soleimani et al., 2019; Manochehrneya et al., 2020; Kalankesh et al., 2022; Leili et al., 2021 ; Sepandi et al., 2021; Sokoty et al., 2021; Sadeghimoghaddam et al., 2021; Aghababaeian et al., 2023 |
| **China** | 11 | Kinay et al., 2019; Teng et al., 2015; Jiang et al., 2020; Li et al., 2020; Yan-Ru et al., 2022; Chan; Ng, 2011; Yang et al., 2005; Chang et al., 2005; Chan et al., 2008; Huang et al., 2017; Chen et al., 2017 |
| **Spain** | 3 | Weilnhammer et al., 2021, Linares; Díaz, 2008  Rodríguez et al., 2022 |
| **Australia** | 2 | Kathleen et al., 2017; Ryan et al., 2015 |
| **Burkina Faso** | 2 | Gottieb-Sthoh et al., 2021; Kynast-Wolf et al., 2010 |
| **South Africa** | 2 | ADEBAYO-OJO et al., 2022; LOKOTOLA et al., 2020 |
| **Brazil** | 1 | Ye et al., 2021. |
| **Chile** | 1 | Sandoval et al., 2021; Román et al., 2009. |
| **Colombia** | 1 | Weinrich et al., 2000. |
| **Pakistan** | 1 | Malik; Khan, 2012. |
| **Other countries** | 15 | Akapinar-Elci et al., 2021; Alahmad et al., 2023; Bertheau et al., 2011; Huang et al., 2021; Huang et al., 2022; Marlier et al., 2013; Mittermeier et al., 2021; Rocque et al., 2021; Van Daalen et al., 2022; Vered et al., 2020; Xu et al., 2020; Martinez-Lozano et al., 2023; Nhung et al., 2023; Armenian et al., 1998; Elkadhi, et al., 2014 |

**Table S3: Characterization of studies by study design**

| **Study designs** | **Number of studies** |
| --- | --- |
| Quantitative studies | 19 |
| Time series | 14 |
| Retrospective studies | 7 |
| Systematic reviews | 7 |
| Narrative reviews | 6 |
| Ecological studies | 5 |
| Literature reviews | 4 |
| Qualitative studies | 4 |
| Cross-sectional studies | 2 |
| Cohort studies | 1 |
| Regression analysis studies | 1 |
| Cross-case studies | 1 |
| Reports | 1 |

**Table S4: Study sources: databases and portals**

| **Databases and portals** | **Number of studies** |
| --- | --- |
| PUBMED/MEDLINE | 28 |
| SCOPUS | 24 |
| EBSCO | 17 |
| SCIELO | 2 |
| BVS | 1 |
| Embase | 1 |
| Institute of Electrical and Electronic Engineers | 1 |


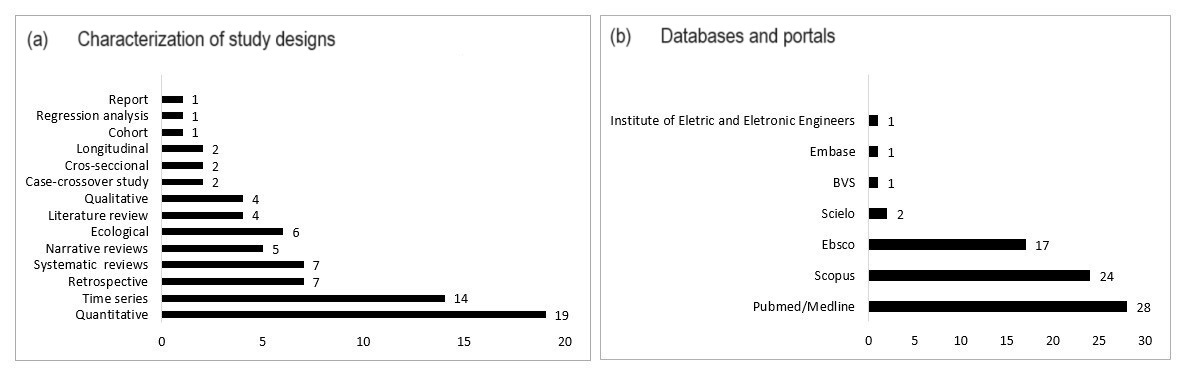


**Figure S1: Distribution of study designs identified in the review, databases and portals**

**Table S5: Studies by diseases associated with climatic variables**

| **Diseases** | **Number of studies** | **Citations** |
| --- | --- | --- |
| Cardiovascular diseases in general | 25 | Achakulwisut et al., 2019; Adbayo-Ojo et al., 2022; Aghababaeian et al., 2021; Berman et al., 2017; Bertheau et al., 2011; Besancenot, 2015; Chan; Ng, 2011; Dahmardeh, 2016; Hua et al., 2021; Kynast-Wolf et al.,2010; Far Away; Green, 2008; Malik; Khan, 2012; Marlier et al., 2013; Mittermeier et al., 2021; Rocque et al., 2021; Van Daalen et al., 2022; Weilnhammer et al., 2021; Elkadhi et al., 2014; Resnick et al., 2015; Ghanizadeh et al., 2015; Soleimani et al., 2019; Sepandi et al., 2021; Ye et al., 2021; Aghababaeian et al., 2023; Nhung et al., 2023 |
| Acute myocardial infarction | 20 | Achakulwisut et al., 2018;Alahmad et al., 2023; Franchini, 2015; Huang et al., 2011; Khraishah et al., 2022; Miller, 2008; Peters et al., 2014; Sandoval et al., 2021; Teng et al., 2015; Román et al., 2009; Soleimani et al., 2019; Quast et al., 2019; Zunnunov, 1991; Chan et al., 2008; Li et al., 2020; Xu et al., 2020; Yan-Ru et al., 2022; Leili et al., 2021; Sokoty et al., 2021; Rawal et al., 2023 |
| Diabetes | 12 | Bell et al., 2022; Cefalu et al., 2006; Gohardehi; Moslehi, 2020; Hua et al., 2021; Kathleen et al., 2017; Miller, 2008; Quart et al., 2019; Armenian et al., 1998; Ryan et al., 2015; Quast et al., 2019; XU et al., 2020; Martinez-Lozano et al., 2023 |
| Stroke | 13 | Alahmad et al., 2023; Croocks et al., 2016; Huang et al., 2022; Khraishah et al., 2022; Yang et al., 2005; Román et al., 2009; Ebrahimi et al., 2014; Quast et al., 2019; Chan et al., 2008; Vered et al., 2020; Yan-Ru et al., 2022; Sokoty et al., 2021; Sadeghimoghaddam et al., 2021 |
| Hypertension | 8 | Gohardehi; Moslehi, 2020; Gottieb-Sthoh et al., 2021; Hua et al., 2021; Weinrich et al., 2000: Armenian et al., 1998; Roman et al., 2009; Quast et al., 2019; Sokoty et al., 2021 |
| Heart failure | 3 | Alahmad et al., 2023; Bell et al., 2022; Khraishah et al., 2022 |

**Table S6: Studies by vulnerable populations**

| **Population** | **Number of studies** | **Citations** |
| --- | --- | --- |
| Older adults | 34 | Achakulwisut et al., 2018; Achakulwisut et al., 2019; Adbayo-Ojo et al., 2022; Aghababaeian et al., 2021; Akapinar-Elci et al., 2021; Bell et al., 2022; Berman et al., 2017; Besancenot, 2015; Chan; Ng, 2011; Cruz-Cano; Mead, 2019; Gottieb-Sthoh et al., 2021; Hua et al., 2021; Kinay et al., 2019; Kynast-Wolf et al., 2010; Far Away; Green, 2008; Malik; Khan, 2012; Peters et al., 2014; Sandoval et al., 2021; Teng et al., 2015; Van Daalen et al., 2022; Armenian et al., 1998; Resnick et al., 2015; Soleimani et al., 2019; Zunnunov, 1991; Lokotola et al., 2020; Vered et al., 2020; Rodríguez et al., 2022; Rahman et al., 2022; Sepandi et al., 2021; Ye et al., 2021; Sadeghimoghaddam et al., 2023; Martinez-Lozano et al., 2023; Nhung et al., 2023 |
| Women | 9 | Adebayo-Ojo et al., 2021; Akpinar-Elci et al., 2021; Gottieb-Sthoh et al., 2021; Quart et al., 2019; Ryan et al., 2015; Quast et al., 2019 |
| Children | 5 | Besancenot, 2015; Cruz-Cano; Mead, 2019; Van Daalen et al., 2022 |

**Table S7: Studies involving older adults with high blood pressure**

| **Older adults with high blood pressure** | |
| --- | --- |
| **Number of studies** | **Citations** |
| **3** | Bell et al., 2022; Gohardehi; Moslehi, 2020; Van Daalen et al., 2022 |


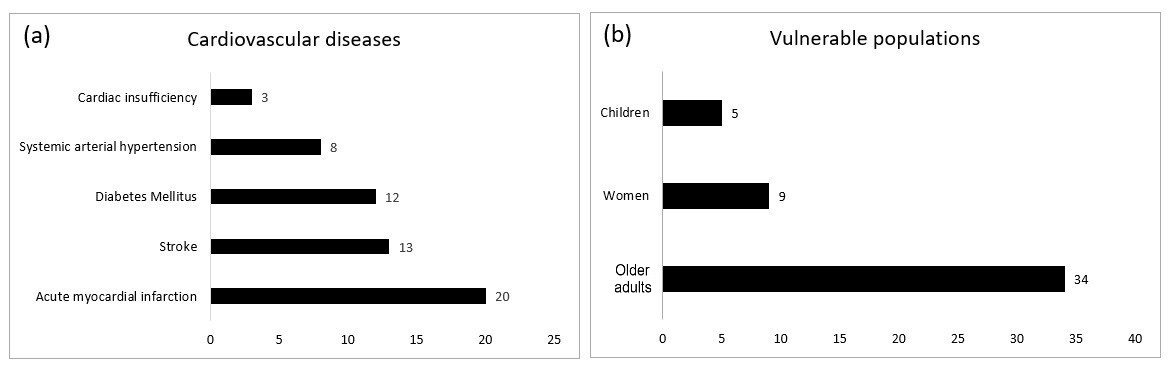


**Figure S2: Distribution of cardiovascular diseases and vulnerable populations identified in the studies**

**Table S8: Studies by climate variable type**

| **Variable** | **Number of studies** | **Citations** |
| --- | --- | --- |
| **Air temperature** | 42 | Aghababaeian et al., 2021; Adebayo-Ojo et al., 2022; Alahmad et al., 2023; Bertheau et al., 2011; Besancenot, 2015; Chan; Ng, 2011; Croocks et al., 2016; Franchini, 2015; Friel et al., 2011; Huang et al., 2011; Khraishah et al., 2022; Kinay et al., 2019; Kynast-Wolf et al., 2010; Malik; Khan, 2012; Mittermeier et al., 2021; Poumadère et al., 2005; Rocque et al., 2021; Sandoval et al., 2021; Teng et al., 2015; Van Daalen et al., 2022; Román et al., 2009; Resnick et al., 2015; Soleimani et al., 2019; Leili et al., 2021; Sepandi et al., 2021; Sokoty et al., 2021; Sadeghimoghaddam et al., 2021; Aghababaeian et al., 2023; Zunnunov, 1991; Chang et al., 2005 ; Linares; Díaz, 2008; Chen et al., 2017; Huang et al., 2017; Li et al., 2017; Jiang et al., 2020; Lokotola et al., 2020; Vered et al., 2020; Xu et al., 2020; Manochehrneya et al., 2020; Yan-Ru et al., 2022; Rodríguez et al., 2022; Rahman et al., 2022 |
| **Air humidity** | 20 | Adbayo-Ojo et al., 2022; Aghababaeian et al., 2021; Croocks et al., 2016; Dahmardeh, 2016; Franchini, 2015; Rocque et al., 2021; Wang et al., 2013; Yang et al. , 2005; Román et al., 2009; Soleimani et al., 2019; Leili et al., 2021; Sepandi et al., 2021; Sokoty et al., 2021; Sadeghimoghaddam et al., 2021; Nhung et al., 2023; Huang et al., 2017; Lokotola et al., 2020; Xu et al., 2020; Manochehrneya et al., 2020; Rodríguez et al., 2022 |
| **Wind speed** | 7 | Akapinar-Elci et al., 2021; Chan, 2011; Longo; Green, 2008; Teng et al., 2015; Weilnhammer et al., 2021; Ebrahimi et al., 2014; Soleimani et al., 2019 |
| **Precipitation** | 3 | Croocks et al., 2016; Malik; Khan, 2012; Kinay et al., 2019 |
| **El Niño** | 1 | Marlier et al., 2013 |
| **Not specified** | 22 | Cruz-Cano; Mead, 2019; Huang et al., 2022; Miller, 2008; Berman et al., 2017; Pachakulwisut et al., 2018; Achakulwisut et al., 2018; Hua et al., 2021; Bell et al., 2022; Cefalu et al., 2006; Quast et al., 2019; Kathleen et al., 2017; Peters et al., 2014; Gohardehi; Moslehi, 2020; Gottieb-Sthoh et al., 2021; Kalankesh et al., 2022; Armenian et al., 1998; Ebrahimi et al., 2014; Rayan et al., 2015; Quast et al., 2019; Ye et al., 2021; Martinez-Lozano et al., 2023; Rawal et al., 2023 |

**Table S9: Studies on the relationship between meteorological variables and CVD and DM**

| **Climate variable** | **Relationship with CVD** | **Citations** |
| --- | --- | --- |
| **Temperature** | - Hot and cold temperatures;  - Heat waves and air pollution;  - Increased temperature: stress and exhaustion, with risk of CVD. | Aghababaeian et al., 2021; Bertheau et al., 2011; Besancenot, 2015; Franchini, 2015; Friel et al., 2011; Huang et al., 2021; Khraishah et al., 2022; Kinay et al., 2019 ; Rocque et al., 2021; Weilnhammer et al., 2021 |
| **Air humidity** | - Low air humidity is related to atmospheric pollution and dust storms;  - The dustiest days are the most polluted days;  - Suspension of atmospheric pollutants: increased cardiovascular risk; | Crooks et al., 2016; Yang et al., 2015 |
| **Precipitation** | - Increased precipitation: Increased CVD | Malik; Awan; Khan, 2012 |
| **Wind** | - Occurrence of disasters: hurricanes  - Favors dust storms and air pollution;  - Greater cardiovascular risk; | Chan; Ng, 2011 |
| **El Niño** | - Forest fires: emission of polluting gases;  - Observed in periods of drought: increase in deaths and hospitalizations due to cardiovascular causes; | Marlier et al., 2013 |

**Table S10: Studies on the relationship between air pollutants and cardiovascular diseases**

| **Type of pollutant** | **Number of studies** | **Citations** |
| --- | --- | --- |
| **PM10** | 15 | Croocks et al., 2016; Chan; Ng, 2011; Akpinar-Elci et al., 2021; Kinay et al., 2019; Aghababaeian et al., 2021; Yang et al., 2005; Achakulwisut et al., 2019 ; Aghababaeian et al., 2021; Adebayo-Ojo et al., 2022; Sokoty et al., 2021; Morris, 2021; Chang et al., 2005; Kalankesh et al., 2022; Rodríguez et al., 2022; Soleimani et al., 2019 |
| **PM2.5** | 15 | Croocks et al., 2016; Chan; Ng, 2011; Khraishah et al., 2022; Achakulwisut et al., 2018; Achakulwisut et al., 2019; Marlier et al., 2013; Aghababaeian et al., 2021; Resnick et al., 2015; Leili et al., 2021; Sepandi et al., 2021; Ye et al., 2021; Morris, 2021; Manochehrneya et al., 2020; Rodríguez et al., 2022; Rahman et al., 2022 |
| **O3** | 9 | Croocks et al., 2016; Chan; Ng, 2011; Khraishah et al., 2022; Marlier et al., 2013; Elkadhi, et al., 2014; Sepandi et al., 2021; Chang et al., 2005; Kalankesh et al., 2022; Soleimani et al., 2019 |
| **NO2** | 9 | Khraishah et al., 2022; Teng et al., 2015; Sepandi et al., 2021; Sokoty et al., 2021; Chang et al., 2005; Huang et al., 2017; Kalankesh et al., 2022; Rodríguez et al., 2022; Soleimani et al., 2019 |
| **SO2** | 8 | Longo; Green, 2008; Chan; Ng, 2011; Khraishah et al., 2022; Elkadhi, et al., 2014; Sepandi et al., 2021; Sokoty et al., 2021; Kalankesh et al., 2022; Soleimani et al., 2019 |
| **CO** | 4 | Sepandi et al., 2021; Sokoty et al., 2021; Chang et al., 2005; Soleimani et al., 2019 |
| **CO2** | 3 | Khraishah et al., 2022; Bertheau et al., 2011; Rodriguez et al., 2011 |
| **Not specified** | 1 | Huang et al., 2021 |

**Table S11: Studies mentioning natural disasters**

| **Type of natural disaster** | **Number of studies** | **Citations** |
| --- | --- | --- |
| Earthquake | 4 | Miller; Arquilla, 2008; Gohardehi; Moslehi, 2020; Armenian et al., 1998; Huang et al., 2022 |
| Hurricane | 12 | Cruz-Cano; Meade, 2019; Mattei et al., 2019; Miller; Arquilla, 2008; Bell et al., 2022; Cefalu et al., 2006; Weinrich et al., 2000; Hua et al., 2021; Quast et al., 2019; Peters et al., 2014; Gohardehi; Moslehi, 2020; Martinez-Lozano et al., 2023; Rawal et al., 2023 |
| Dust storms | 14 | Croocks et al., 2016; Chan; Huey, 2011; Akpinar-Elci et al., 2021; Aghababaeian et al., 2021; Yang et al., 2005; Achakulwisut et al., 2019; Aghababaeian et al., 2021 ; Teng et al., 2015; Sadeghimoghaddam et al., 2021; Ebrahimi et al., 2014; Ryan et al., 2015; Zunnunov, 1991; Chan et al., 2008; Li et al., 2020 |
| Fires | 5 | Sandoval et al., 2021; Gohardehi; Moslehi, 2020; Ye et al., 2021; Resnick et al., 2015; Marlier et al., 2013 |
| Heat waves | 4 | Franchini, 2015; Linares; Díaz, 2008; Xu et al., 2020; Nhung et al., 2023 |
| Unspecified disasters | 2 | Huang et al., 2021; Malik; Khan, 2012 |

**
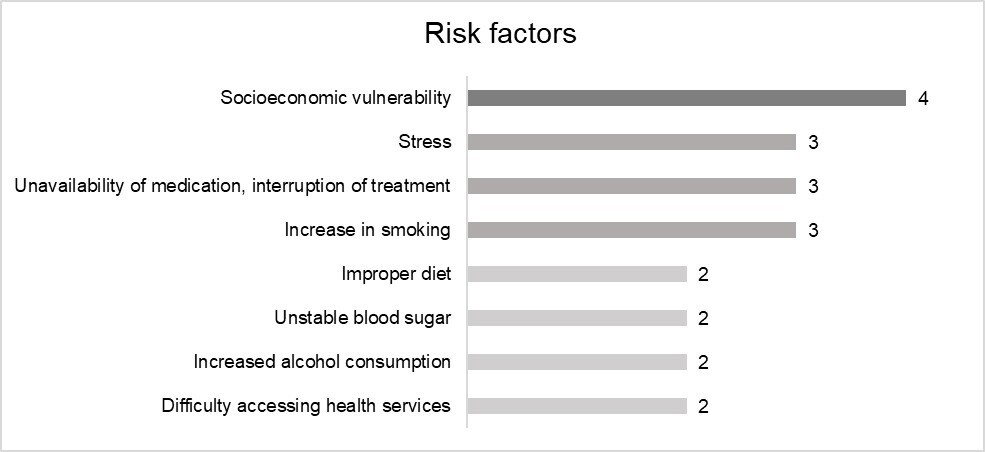
**

**Figure S3: Distribution of risk factors identified in the studies**

**Table S12: Studies on risk factors associated with climate variables and diseases**

| **Risk factors** | **Number of studies** | **Citations** |
| --- | --- | --- |
| Exposure to air pollutants | 30 | Achakulwisut et al., 2018; Achakulwisut et al., 2019; Adebayo-Ojo et al., 2022; Aghababaeian et al., 2021; Akpinar-Elci et al., 2021; Bertheau et al., 2011; Chan, 2011 ; Croocks et al., 2016; Huang et al., 2021; Khraishah et al., 2022; Kinay et al., 2019; Longo; Green, 2008; Marlier et al., 2013; Salvador et al., 2019; Teng et al., 2015; Aghababaeian et al., 2021; Yang et al., 2005; Elkadhi, et al., 2014; Resnick et al., 2015; Soleimani et al., 2019; Morris, 2021; Chang et al., 2005; Manochehrneya et al., 2020; Kalankesh et al., 2022; Rodríguez et al., 2022; Rahman et al., 2022; Leili et al., 2021; Sepandi et al., 2021; Sokoty et al., 2021; Ye et al., 2021 |
| Exposure to extreme temperatures | 19 | Alahmad et al., 2023; Besancenot, 2015; Franchini, 2015; Huang et al., 2021; Huang et al., 2022; Malik; Khan, 2012; Mittermeier et al., 2021; Rocque et al., 2021; Resnick et al., 2015; Chang et al., 2005; Linares; Díaz, 2008; Chen et al., 2017; Li et al., 2017; Jiang et al., 2020; Lokotola et al., 2020; Vered et al., 2020; Yan -Ru et al., 2022; Rahman et al., 2022; Aghababaeian et al., 2023 |
| Exposure to natural disasters | 16 | Cruz-Cano; Mead, 2019; Gohardehi; Moslehi, 2020; Huang et al., 2021; Huang et al., 2022; Malik; Khan, 2012; Marlier et al., 2013; Mattei et al., 2022; Miller; Arquilla, 2008; Quart et al., 2019; Sandoval et al., 2021; Weinrich et al., 2000; Armenian et al., 1998; Resnick et al., 2015; Ye et al., 2021; Martinez-Lozano et al., 2023; Rawal et al., 2023 |
| Heat waves | 4 | Franchini, 2015; Linares; Díaz, 2008; Xu et al., 2020; Nhung et al., 2023 |
| Hypertension | 4 | Huang et al., 2021; Huang et al., 2022; Kathleen et al., 2017; Rawal et al., 2023 |
| Socioeconomic vulnerability | 4 | Akpinar-Elci et al., 2021; Alahmad et al., 2023; Huang et al., 2021; Malik; Khan, 2012 |
| Stress | 3 | Cefalu et al., 2006; Hua et al., 2021; Quart et al., 2019 |
| Unavailability of medication, non-adherence and interruption of treatment | 3 | Cefalu et al., 2006; Hua et al., 2021; Kathleen et al., 2017 |
| Smoking | 3 | Huang et al., 2021; Huang et al., 2022; Peters et al., 2014 |
| Poor diet | 2 | Elkadhi, et al., 2014; Quart et al., 2019 |
| Chronic diseases | 2 | Bell et al., 2022; Gohardehi; Moslehi, 2020 |
| Unstable blood sugar | 2 | Huang et al., 2021; Huang et al., 2022 |
| Residents in urban areas | 2 | Kinay et al., 2019; Van Daalen et al., 2022 |
| High cholesterol levels | 2 | Huang et al., 2022; Kathleen et al., 2017 |
| Alcoholism | 2 | Huang et al., 2021; Peters et al., 2014 |
| Difficulty accessing health services | 2 | Huang et al., 2022; Peters et al., 2014 |
| Exposure to seasonality | 2 | Gottieb-Sthoh et al., 2021; Kynast-Wolf et al., 2010. |
| Increase in BMI | 1 | Huang et al., 2021 |
| Physically active individuals (dehydration risk) | 1 | Van Daalen et al., 2022 |
| Sedentary lifestyle | 1 | Huang et al., 2021 |
